# Pan-cancer analyses of senescence-related genes in extracellular matrix characterization in cancer

**DOI:** 10.1007/s12672-023-00828-7

**Published:** 2023-11-20

**Authors:** Bo Yan, Pan Liao, Liqiu Shi, Ping Lei

**Affiliations:** 1 Haihe Laboratory of Cell Ecosystem, Department of Geriatrics, Tianjin Medical University General Hospital, 154 Anshan Road, Heping District, Tianjin, 300052 China; 2 Tianjin Geriatrics Institute, Tianjin Medical University General Hospital, 154 Anshan Road, Heping District, Tianjin, 300052 China; 3 The School of Medicine, Nankai University, 94 Weijin Road, Tianjin, 300071 China; 4 Inner Mongolia Forestry General Hospital, 81 Lincheng North Road, Yakeshi, 022150 Inner Mongolia China

**Keywords:** Pan-cancer, ECM-SRGs, Genomics, Immunogenomic, Epigenetic, Prognosis

## Abstract

**Purpose:**

The aged microenvironment plays a crucial role in tumor onset and progression. However, it remains unclear whether and how the aging of the extracellular matrix (ECM) influences cancer onset and progression. Furthermore, the mechanisms and implications of extracellular matrix senescence-related genes (ECM-SRGs) in pan-cancer have not been investigated.

**Methods:**

We collected profiling data from over 10,000 individuals, covering 33 cancer types, 750 small molecule drugs, and 24 immune cell types, for a thorough and systematic analysis of ECM-SRGs in cancer.

**Results:**

We observed a significant correlation between immune cell infiltrates and Gene Set Variation Analysis enrichment scores of ECM-SRGs in 33 cancer types. Moreover, our results revealed significant differences in immune cell infiltration among patients with copy number variations (CNV) and single nucleotide variations (SNV) in ECM-SRGs across various malignancies. Aberrant hypomethylation led to increased ECM-SRGs expression, and in specific malignancies, a connection between ECM-SRGs hypomethylation and adverse patient survival was established. The frequency of CNV and SNV in ECM-SRGs was elevated. We observed a positive correlation between CNV, SNV, and ECM-SRGs expression. Furthermore, a correlation was found between the high frequency of CNV and SNV in ECM-SRGs and poor patient survival in several cancer types. Additionally, the results demonstrated that ECM-SRGs expression could serve as a predictor of patient survival in diverse cancers. Pathway analysis unveiled the role of ECM-SRGs in activating EMT, apoptosis, and the RAS/MAPK signaling pathway while suppressing the cell cycle, hormone AR, and the response to DNA damage signaling pathway. Finally, we conducted searches in the “Genomics of Drug Sensitivity in Cancer” and “Genomics of Therapeutics Response Portal” databases, identifying several drugs that target ECM-SRGs.

**Conclusions:**

We conducted a comprehensive evaluation of the genomes and immunogenomics of ECM-SRGs, along with their clinical features in 33 solid tumors. This may provide insights into the relationship between ECM-SRGs and tumorigenesis. Consequently, targeting these ECM-SRGs holds promise as a clinical approach for cancer treatment.

**Supplementary Information:**

The online version contains supplementary material available at 10.1007/s12672-023-00828-7.

## Introduction

Aging is a progressive degenerative condition marked by tissue stem cell depletion, inflammation, matrix changes, cellular senescence, and metabolic dysfunction [1]. Aging is a natural process that occurs over time and shares common features with cancer. It is also a significant risk factor for the onset of cancer [2], suggesting that aging may promote tumorigenesis. This clinical observation has been attributed to the influence of aging on cellular genetics [3]. As a result, studies of age-related changes in tissues have typically focused on the cellular level [4, 5]. In recent years, there has been significant interest in the interaction between tumor cells and the tumor microenvironment (TME) in the progression of malignant tumors [6, 7]. As a critical component of the TME, the extracellular matrix (ECM) primarily consists of collagen, fibronectin, laminin, glycosaminoglycans, proteoglycans, and various ECM remodeling enzymes. Its primary role is to provide vital biochemical and biomechanical support for the resident cells. ECM remodeling, characterized by substantial changes in ECM composition and organization, has been closely linked to tumor differentiation, proliferation, and metastasis [8, 9]. An investigation of ECM gene dysregulation across various cancer types revealed a subset of ECM genes that are specifically dysregulated in tumors. High expression of these genes was associated with an unfavorable prognosis in pan-cancer analyses [10]. Extensive ECM changes have been observed in breast cancer, such as the upregulation and rearrangement of fibrillar collagen, fibronectin, and other remodeling enzymes, leading to increased ECM stiffness [11–13]. Extensive clinical imaging and pathological evidence also support the idea that the density and hardness of tumor tissue are closely linked to its malignancy [14]. Additionally, ECM remodeling can lead to the overexpression of receptors such as EGFR, ERBB2, CD44, and others in the TME. This, in turn, can induce tumor invasion and metastasis by activating downstream signaling pathways like PI3K/Akt and MAPK [15–17]. The influence of age-related dysregulation of cellular processes on carcinogenesis is widely recognized. However, alterations in the extracellular matrix (ECM) and other changes in the microenvironment are frequently overlooked. It is a well-established fact that when cells are transplanted into the livers of elderly rats, as opposed to young ones, rat liver epithelial cells that have undergone neoplastic transformation exhibit increased rates of tumor formation [18]. This suggests that the aged microenvironment plays a critical role in tumor development and dissemination. Nonetheless, it remains unclear whether and how the aging of the ECM impacts the initiation and progression of cancer.

Even minor variations in the biochemical composition, rigidity, and structure of the ECM can lead to a significant alteration in cellular responsiveness [19–21]. Collagen production decreases, and the ECM loses its integrity in the aged microenvironment, thereby enhancing the invasive potential of tumor cells [2]. Another age-related alteration in the ECM is the thinning of fibers, which can promote metastasis [22]. Conversely, the transformation of normal breast epithelial cells into tumor precursors necessitates a rigid substrate [23], and cancer cells inhibit the development and maturation of adipocytes on stiff matrices [24]. Additionally, when malignant progenitors are cultured on soft substrates, they revert to a normal epithelial cell state [25].

We performed a comprehensive analysis of the genetic, immunological, and clinical characteristics of 19 ECM-senescence-related genes (ECM-SRGs) across 33 cancer types. This is due to a lack of research on age-related ECM changes in cancer. Our findings revealed that genomic, epigenetic alterations, and immunogenomic changes in ECM-SRGs are associated with their abnormal expression. Moreover, a notable correlation was observed between the abnormal expression of ECM-SRGs, the activation of cancer-related pathways, and patient survival. Therefore, the development of strategies targeting these ECM-SRGs may hold promise for the treatment of cancer patients.

## Materials and methods

### Dataset and tumor types

Changes in the genetic and immune microenvironment play a role in regulating tumorigenesis, cancer progression, diagnosis, prognosis, and treatment outcomes for patients. In this era of abundant biological data, individual gene expression can be obscured by significant background interferences. Yet, a combination of gene sets or gene scores from multiple patients at each stage of tumorigenesis, obtained from various databases, can offer insights into the underlying processes related to cancer. Consequently, we conducted an analysis of genetic alterations, including gene expression, copy number variations (CNV), single nucleotide variations (SNV), and gene methylation status. We also assessed patient sensitivity to drugs and the infiltration profiles of 24 types of immune cells [26].

Data on immune cell infiltration (ICI, n = 10,995), gene expression (n = 10,995), clinical features (n = 11,160), cancer staging (n = 9478), copy number variations (CNV, n = 11,495), and methylation (450 k level 3) of patients were extracted from “The Cancer Genome Atlas” (TCGA) database using UCSC Xena (http://xena.ucsc.edu/). Subsequently, we acquired SNV data (n = 10,234) from Synapse (syn7824274; https://www.synapse.org/#!Synapse:syn7824274) and reverse-phase protein array (RPPA) data (n = 7,876) from “The Cancer Proteome Atlas” (TCPA; https://tcpaportal.org/tcpa/index.html) database. Lastly, we retrieved data from “The Genomics of Drug Sensitivity in Cancer” (GDSC; https://www.cancerrxgene.org/) and “Cancer Therapeutics Response Portal” (CTRP; https://portals.broadinstitute.org/ctrp/) databases to investigate the relationship between ECM-SRGs expression and patients’ responsiveness to drugs. We conducted a pan-cancer analysis using data from patients with 33 different cancer types, including acute myeloid leukemia (LAML), adrenocortical carcinoma (ACC), bladder urothelial carcinoma (BLCA), breast invasive carcinoma (BRCA), cervical squamous cell carcinoma and endocervical adenocarcinoma (CESC), cholangiocarcinoma (CHOL), colon adenocarcinoma (COAD), esophageal carcinoma (ESCA), glioblastoma (GBM), head and neck squamous cell carcinoma (HNSC), kidney chromophobe (KICH), kidney renal clear cell carcinoma (KIRC), kidney renal papillary cell carcinoma (KIRP), lower grade glioma (LGG), liver hepatocellular carcinoma (LIHC), lung adenocarcinoma (LUAD), lung squamous cell carcinoma (LUSC), lymphoid neoplasm diffuse large B-cell lymphoma (DLBC), mesothelioma (MESO), ovarian serous cystadenocarcinoma (OV), pancreatic adenocarcinoma (PAAD), pheochromocytoma and paraganglioma (PCPG), prostate adenocarcinoma (PRAD), rectum adenocarcinoma (READ), sarcoma (SARC), skin cutaneous melanoma (SKCM), stomach adenocarcinoma (STAD), testicular germ cell tumors (TGCT), thymoma (THYM), thyroid carcinoma (THCA), uterine carcinosarcoma (UCS), uterine corpus endometrial carcinoma (UCEC), and uveal melanoma (UVM). We also examined gene signatures for 24 types of immune cells. Senescent cells are identified through a novel gene set, which also predicts senescence-related processes within tissues [27]. Additionally determined depending on gene ontology terms and were presented in Table S3.Subsequently, we identified 328 ECM-associated genes based on gene ontology terms and documented them in Table S3. Ultimately, we identified 19 ECM-SRGs (Fig. S1).

### ICI and gene set variation analysis (GSVA)

We examined the association between ECM-SRG expression and Immune Cell Infiltration (ICI) using the ICI & GSVA score module and assessed the infiltration of 24 types of immune cells using ImmuCellAI (http://bioinfo.life.hust.edu.cn/ImmuCellAI/#!/). Integrated gene set expression levels and gene set expressions are positively correlated with the GSVA score. Patients in the tumor group (TG) with high GSVA scores generally exhibited higher gene set expression compared to the adjacent group. The GSVA score (http://bioinfo.life.hust.edu.cn/GSCA/#/) was calculated using the “GSVA” function in the R program. Subsequently, in order to establish the relationship between ECM-SRG expression and ICI, Spearman correlation analysis was performed to calculate a correlation coefficient with FDR-adjusted p-values. Gene set signatures were employed to estimate the abundance of 24 distinct types of immune cells (as documented). Overlapping genes between input and signature were excluded during ICI estimation.

### ICI and SNV

We utilized the ICI & SNV module to investigate the correlation between Immune Cell Infiltration (ICI) and Single Nucleotide Variations (SNV) in ECM-SRGs. ImmuCellAI was employed to assess the infiltration of 24 distinct types of immune cells. The comprehensive SNV status of the input genes in all samples is denoted by “SNV” in the gene set. Patients with a mutation in at least one gene from the input gene set would be classified as belonging to the mutant group (MG). Subsequently, patients without SNVs in any of the genes from the gene set were classified as belonging to the wild-type (WT) group. Lastly, we conducted a Wilcoxon test to examine the association between ICI and SNV in ECM-SRGs by comparing the mean infiltration of the gene set SNV group. With False Discovery Rate (FDR) adjustment for p-values.

### ICI and CNV

We examined the correlation between Immune ICI and Copy Number Variations (CNV) in ECM-SRGs using the ICI & CNV module and evaluated the infiltration status of 24 distinct types of immune cells using ImmuCellAI. The overall CNV status of the input gene set for all patients is represented by the integrated CNV status of the gene sets. Subsequently, patients were categorized into Amplification (Amp) or Deletion (Dele) groups based on whether they had amplifications or deletions in at least one gene within the gene set. Patients in the WT group had no CNVs in any of the genes in the gene set. Patients with inconsistencies in gene CNV, such as amplification of gene A while deletion of gene B in sample 1, were categorized as belonging to the Excluded group. Ultimately, we conducted either the Wilcoxon test for two-group comparisons or one-way ANOVA for more than two groups to compare the mean ICI in samples and determine the correlation between ICI and CNV in ECM-SRGs. FDR was employed to adjust the P-values.

### Differential GSVA

In an unsupervised approach, GSVA was employed to evaluate variations in gene set expression, which is represented as the GSVA score, among patients with specific cancers. This module generates a differential GSVA score in both patient and normal samples. The gene set expression is positively correlated with the GSVA score, which represents the comprehensive gene set expression. The expression level of the entire gene set was high in patients in the TG if their GSVA scores exceeded those of the adjacent group. The GSVA scores were calculated using the “GSVA” R package.

### Differential gene expression analysis

We obtained data on RNA-Seq (n = 10,995) and clinical characteristics (n = 11,160) of patients from TCGA. For the differential expression analysis, we integrated the normalized and batch-corrected RSEM gene expression data from paired tumor and normal samples. The fold change (FC) was calculated using the formula: mean (Tumor)/mean (Normal). Finally, we performed the t-test to calculate the P-value and applied FDR adjustment.

### Gene-set enrichment analysis (GSEA)

Gene Set Enrichment Analysis (GSEA) is a computational method used to assess the statistical significance of a predefined gene set in the context of differences between two biological states, such as phenotypes. We conducted GSEA using the “fgsea” R package to determine whether an input gene set ranked at the top or bottom of the list based on the FC in gene expression between tumor and normal samples.

### Expression and subtype analysis

Different tumors exhibit distinct gene expression profiles. As a result, we conducted an analysis of gene expression and identified subtypes to ascertain variations in patient gene expression. We integrated gene expression data with the clinical subtype information of patients using the sample barcode. Each subtype consisted of at least five samples. We compared the GSVA scores of patients within subtypes using the Wilcoxon test for two subtypes and ANOVA for more than two subtypes. Subtypes represent molecular subtypes when available; otherwise, they represent clustering subtypes.

### Gene expression and stage analysis

To conduct gene expression and stage analysis, we obtained data on the pathological stages of 9478 patients. Each stage subgroup was required to have a minimum of five samples. We compared gene expression across different groups using the Wilcoxon test for two stage groups and ANOVA for more than two stage groups. Patients were categorized into stages I, II, III, and IV, with each stage further divided as follows: Stage I: I, IA, IB, and IC; Stage II: II, IIA, IIB, and IIC; Stage III: III, IIIA, IIIB, and IIIC; and Stage IV: IV, IVA, IVB, and IVC.

### Gene expression and survival analysis

In the context of gene expression and survival analysis, we initially obtained the clinical data of patients with 33 different cancer types. Patients with unavailable data and those at risk of death from other causes in addition to cancer were excluded from further analysis, particularly for assessing the disease-free interval (DFI) and disease-specific survival (DSS). Next, we integrated gene expression data with patient survival information using sample barcodes. Following this, patients were divided into two groups based on their gene expression values, with one being the high-expression group (HRG) and the other the low-expression group (LRG). We then utilized the “survival” package in the R program to ascertain the duration and status of patient survival in the two groups. Finally, we performed Cox Proportional-Hazard and Logrank tests on all genes in all cancer types. Futhermore, this study adopted ‘survival analysis’ to examine the association of gene expression level with survival of diverse TCGA-derived cancers in GEPIA2 (http://gepia2.cancer-pku.cn/).

### CNV analysis

The CNV summary module provides an overview of copy number variations (CNV) in selected cancer types within input genes. We obtained CNV data from 11,495 patients from TCGA and conducted a screening for significantly amplified or deleted regions in these genes using GISTIC2.0. The GISTIC score represents the level of CNV per gene: a deep loss or homozygous deletion is denoted by − 2, a shallow loss or heterozygous deletion is represented by − 1, a shallow deletion by 0, signifying diploid status. Moreover, a score of one or more indicates a slight increase, such as a gain of a few additional copies, often broad gains, or heterozygous amplification. A score of two or more signifies substantial amplification, potentially involving more local copies or even homozygous amplification. These four categories of CNV in input genes were compiled for various types of cancer (GISTIC scores: − 2, − 1, 1, 2).

Following the procedure outlined by Schlattl et al., we conducted Spearman correlation analysis on this module to establish the connection between CNV and gene expression [28]. We retrieved data on RSEM normalized gene expression and CNV of patients from TCGA, and these datasets were merged based on the TCGA barcode. FDR was used for P-value adjustments.Furthermore, we utilized the CNV & survival module to assess differences in the survival of patients with CNV-altered genes and those with WT genes. We obtained CNV and clinical data for 11,495 patients across 33 different cancer types from TCGA. Patients with unavailable data and those with competing risks of death due to cancer were excluded from subsequent analyses, particularly for DSS and DFI data. The CNV and survival information of patients were merged based on their sample barcodes. Patients were categorized into three groups: WT, Amplified (Amp.), and Deleted (Dele.). We calculated the duration and survival status of patients in these groups using the “survival” R package and conducted Logrank tests to assess differences in survival. A P-value for survival analysis was calculated using groups with two or more samples and groups with less than two samples to construct the survival curve.

Additionally, we analyzed the frequency of genomic alteration types across various cancer types using the Cancer Types Summary module of the online web tool cBioPortal (https://www.cbioportal.org/).

### Methylation analysis

The differential methylation module offers information about the methylation status in patients with cancer and normal samples. Data on Illumina HumanMethylation 450 k level 3 from more than ten paired tumor and adjacent non-tumor samples of patients from TCGA were acquired. Multiple methylation sites exist within a gene, and multiple data tags store information about the methylation level at each site. Initially, we performed a correlation analysis to explore methylation sites that exhibited a negative inverse relationship with gene expression. T-test results were employed to calculate the p-value, which was subsequently adjusted using the FDR. We utilized the Methylation and Expression module to investigate the relationship between methylation levels and gene expression through Spearman correlation analysis. Initially, we obtained data on RSEM-normalized gene expression and Illumina Methylation 450 k level 3 from TCGA. Information regarding patient survival in the high- and low-methylation groups can be found in the methylation & survival module. We obtained Illumina HumanMethylation 450 k level 3 data of patients from 33 cancer subtypes in TCGA and a prior study [29]. Several methylation sites exist in a gene and multiple tags store data on the methylation levels at each site. In order to find methylation sites that are negatively correlated with gene expression, we performed correlation analysis. Patients whose data was not available and those at risk of competing for death due to cancer were removed from subsequent analyses (DSS and DFI data). Methylation and survival data were combined using sample barcodes. Patients were classified into high- and low-methylation groups based on their median methylation levels. We utilized the “survival” tool in the R program to calculate the duration and status of survival for both groups of patients. Additionally, the Hazard Ratio (HR) was calculated using a Cox Proportional-Hazards model, and the Log-rank test was performed to determine if there were differences in survival rates between the two methylation groups.

### SNV analysis

The SNV summary module offers data on the Single Nucleotide Variations (SNV) of input genes in selected cancer types. We acquired SNV data from 10,234 patients across 33 cancer types from TCGA. Deleterious mutations, such as missense, nonsense, frameshift insertions and deletions, splice site mutations, and inframe insertions, were considered for this analysis. Silent mutations, intronic mutations, mutations in intergenic regions (IGR), mutations in 3’ and 5’ untranslated regions (UTR), as well as mutations in 3’ and 5’ Flank regions, were considered non-deleterious. The SNV & survival module offers information on patient survival based on the SNV in genes. Initially, we collected data on SNV and patient survival from TCGA and merged these datasets using sample barcodes. Subsequently, patients with deleterious mutations in specific genes were categorized into the MG (Mutant Group). Additionally, survival analysis was conducted on groups with more than two samples, with a minimum of two groups being necessary. The duration and survival status of patients in both groups were evaluated using the “survival” function in the R program. Lastly, we conducted Cox Proportional-Hazards and Logrank tests to assess the difference in the survival of patients between the WT and MG.

### Analysis of gene expression and GSVA and pathway activity

Variations in GSVA scores and gene expression across pathway activity groups were assessed using the GSVA, gene expression, and pathway activity modules (activation and suppression). Median pathway scores were used for group definition. We utilized RPPA data from TCPA to calculate the activity scores of 10 cancer-related pathways for 7876 patients across 32 cancer types from TCGA. RPPA is a high-throughput antibody-based technique similar to western blotting. Initially, proteins were extracted from cultured cells or tissue samples, denatured with SDS, transferred onto nitrocellulose-coated slides, and subsequently probed with antibodies.

Subsequently, we examined the RTK, TSC/mTOR, PI3K/AKT, apoptosis, RAS/MAPK signaling pathways, cell cycle, ER, AR, epithelial-to-mesenchymal transition (EMT), and response to DNA damage. Lastly, the relative protein expression for all samples was assessed using median-centered RPPA-RBN data and normalized by calculating the standard deviation. The pathway score was calculated using the formula: Σ (the relative protein expression of all positive regulatory components—the negative regulatory components of a specific pathway) [30]. Subsequently, patients were grouped into LRG and HRG based on their median gene expression. Furthermore, the difference in pathway activity score (PAS) between the two groups was determined using a Student’s t-test, and the P-value was adjusted for FDR. FDR ≤ 0.05 was the threshold for significance. Gene A might either activate a pathway depending on whether PAS (High-gene A expression) > PAS (Low-gene A expression) or block a pathway if otherwise, as described previously [30, 31]. The "GSVA" function in the R program, which reveals variations in gene expression among patients with specific cancers in an unsupervised manner, was employed to calculate the GSVA score. For a deeper understanding of gene function and pathways in pan-cancer, we explored various databases. Correlations between genes and their functional states in different cancers were assessed using the CancerSEA database (http://biocc.hrbmu.edu.cn/CancerSEA/).

### Drug sensitivity analysis using GDSC and CTRP

Initially, we acquired the IC_50_ values for 265 small molecules in 860 cell lines along with their gene expression data from GDSC, and for 481 small molecules in 1001 cell lines with their gene expression data from the CTRP. Subsequently, we integrated the information pertaining to gene expression and drug sensitivity in patients. Lastly, we assessed the association between IC_50_ values and gene expression through Pearson correlation analysis, with P-value adjustments performed using FDR.

### Statistical analysis

We performed statistical analysis using the “R” software (version 4.2.1, http://www.r-project.org). We used Spearman correlation analysis to calculate correlation coefficients. To calculate the survival risk and HR of patients, we utilized a Cox proportional hazards model. We compared patients’ GSVA scores across different groups using the Wilcoxon test (for groups with 2 stages) and ANOVA (for groups with more than 2 stages). We performed trend analysis using the Mann–Kendall Trend Test and determined the difference in PAS between the two groups using a Student’s t-test. A significance level was indicated by *P* < *0.05* or *FDR* ≤ *0.05*.

## Results

### ICI analysis

**Fig. 1:**
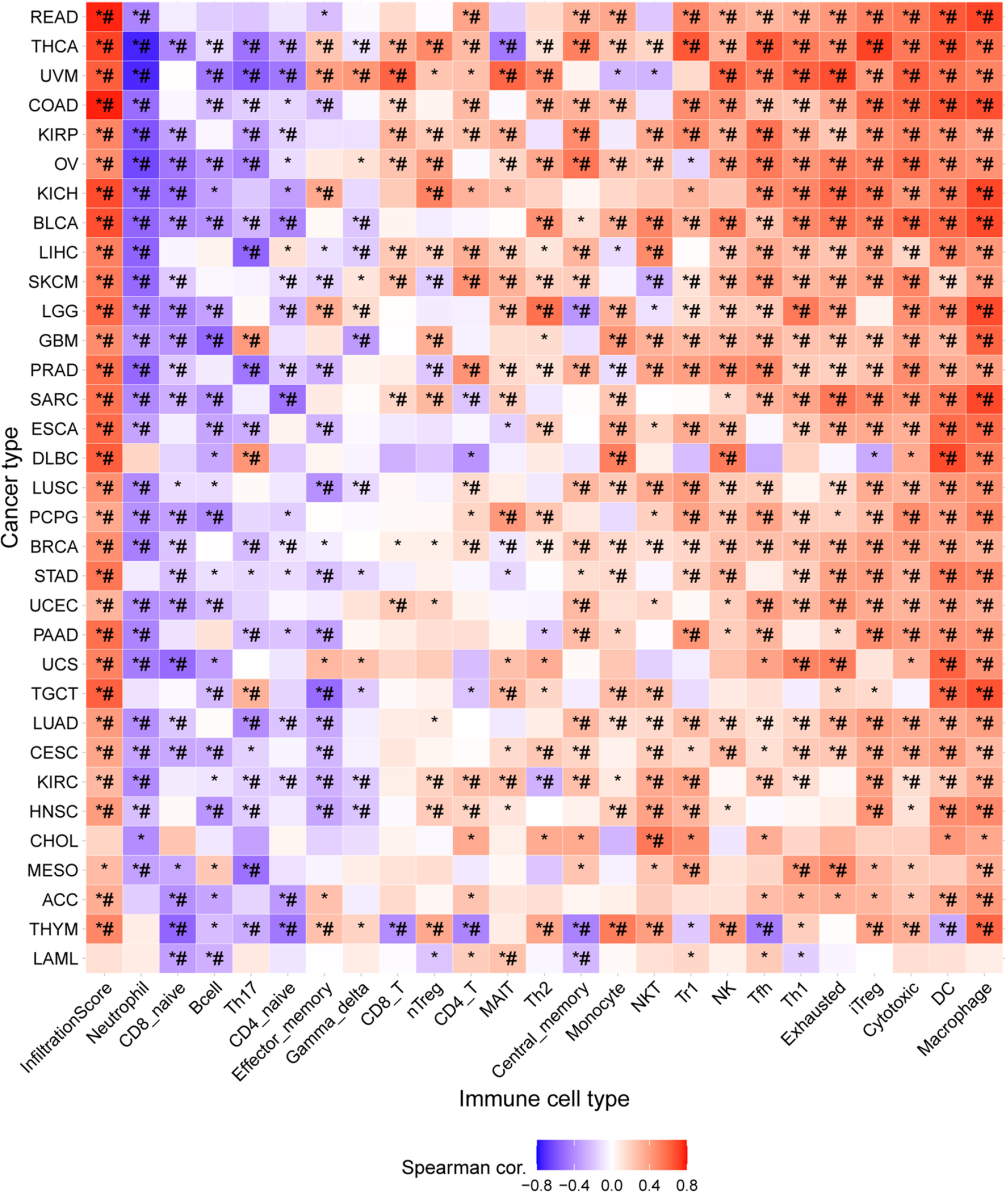
Correlation between gene set expression (GSVA) enrichment scores of extracellular-matrix-senescence-related genes and immune cell infiltration in 33 cancers. *P value ≤ *0.05; *^*#*^* FDR* ≤ *0.05*

Senescent cells are characterized by a unique gene set that also predicts senescence-related processes across various tissues [27]. Subsequently, 328 ECM-associated genes were identified based on gene ontology terms and are listed in Table S3. Lastly, we identified 19 ECM-SRGs (MMP2, SERPINE1, ICAM3, TNF, MMP13, PECAM1, MMP3, LCP1, SPP1, MMP14, TIMP2, TNFRSF11B, FGF2, ITGA2, ICAM1, MMP10, MMP1, MMP12, and MMP9) (Fig. S1). We established a significant correlation between GSVA enrichment scores (ES) of ECM-SRGs and ICI in 33 different cancers (*P-value* and *FDR* ≤ *0.05*, Fig. 1). We also demonstrated a significant correlation between 19 ECM-SRGs and ICI in 33 cancers (Table S4). Moreover, the results showed a positive correlation between GSVA-ES of ECM-SRGs and Macrophage, DCs, iTreg, cytotoxic and exhausted T cell, Th1, Tfh, and NK cells. Additionally, we observed a negative correlation between GSVA-ES of ECM-SRGs and naive CD8 and CD4 T cell, neutrophil, and B cells in pan-cancer (*P*-value and *FDR* ≤ *0.05*, Fig. 1). Conversely, in pan-cancer, GSVA-ES of ECM-SRGs were positively correlated with the InfiltrationScore of patients (*P-value and FDR* ≤ *0.05*, Fig. 1). Thus, aberrant ECM-SRGs expression regulates ICI in patients, thereby indicating a significant role of ECM-SRGs in cancer progression.

**Fig. 2:**
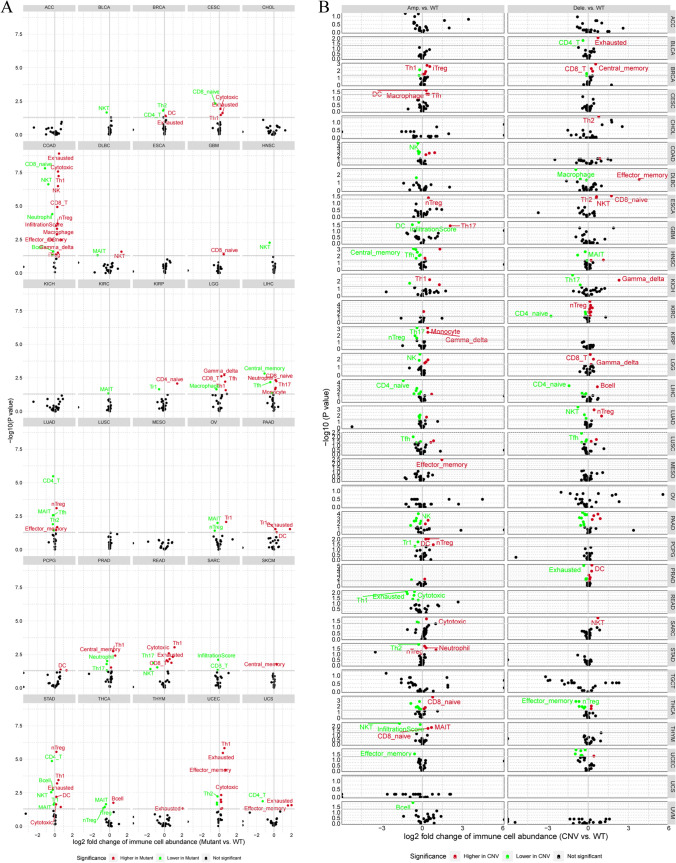
Difference of immune cell infiltration in patients with (**A**) single nucleotide variation and (**B**) copy number variation (CNV) amplification in extracellular-matrix-senescence-related genes. *WT* wild-type

Alterations in the immune microenvironment could be involved in tumorigenesis, cancer progression, diagnosis, prognosis, and therapeutic of patients. Further, a significant difference in ICI in patients harboring SNVs in ECM-SRGs in UCS, UCEC, THYM, THCA, STAD, SKCM, SARC, READ, PRAD, PCPG, PAAD, OV, LUAD, LIHC, LGG, KIRP, KIRC, HNSC, GBM, DLBC, COAD, CESC, BRCA, and BLCA (*FDR* ≤ *0.05*, Fig. 2A and Table S5). A significant correlation between CNV amplification of ECM-SRGs and ICI in UVM, UCEC, THYM, THCA, STAD, SARC, READ, PCPG, PAAD, MESO, LUSC, LIHC, LGG, KIRP, KICH, HNSC, GBM, ESCA, COAD, CESC, and BRCA was observed. Moreover, the results revealed a significant correlation between CNV deletions of ECM-SRGs and ICI in THCA, SARC, PRAD, LUSC, LUAD, LIHC, LGG, KIRC, KICH, HNSC, ESCA, DLBC, CHOL, BRCA, and BLCA (*FDR* ≤ *0.05*, Fig. 2B and Table S6). Moreover, a significant correlation between the methylation of ECM-SRGs and ICI in ECM-SRGs (Table S7).

These findings suggest that abnormalities in ECM-SRGs within the immune microenvironment play a role in cancer initiation, progression, diagnosis, prognosis, and therapeutic outcomes.

### Analysis of ECM-SRGs expression, cancer subtypes, and stages

**Fig. 3:**
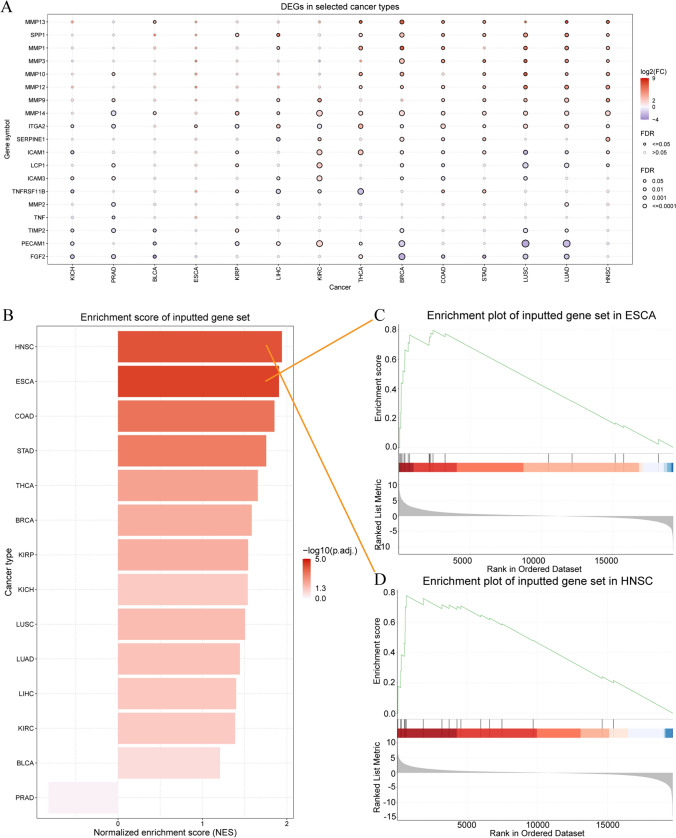
Gene set expression analysis and gene-set enrichment analysis (GSEA) of extracellular-matrix-senescence-related genes (ECM-SRGs). **A** The mRNA differences between normal samples and tumor samples. **B** Enrichment score of ECM-SRGs. **C** Enrichment plot of ECM-SRGs in ESCA. **D** Enrichment plot of ECM -SRGs in HNSC

We assessed differences in ECM-SRGs expression among cancer patients based on the GSVA score. Compared to normal tissue, patients with 13 solid tumors displayed significant differences in the expression of ECM-SRGs (*FDR* ≤ *0.05*, Fig. 3A and Table S8). However, there were no significant differences in the expression of ECM-SRGs among patients with ESCA.

The bar plot displays the GSVA scores of ECM-SRGs compared to all 20,000 + genes (background) in various cancers (Fig. 3B and Table S9). The results indicated elevated expression of most ECM-SRGs in patients with ESCA (Fig. 3C), HNSC (Fig. 3D), COAD, STAD, THCA, BRCA, and KIRP (*P* < *0.05*).

**Fig. 4:**
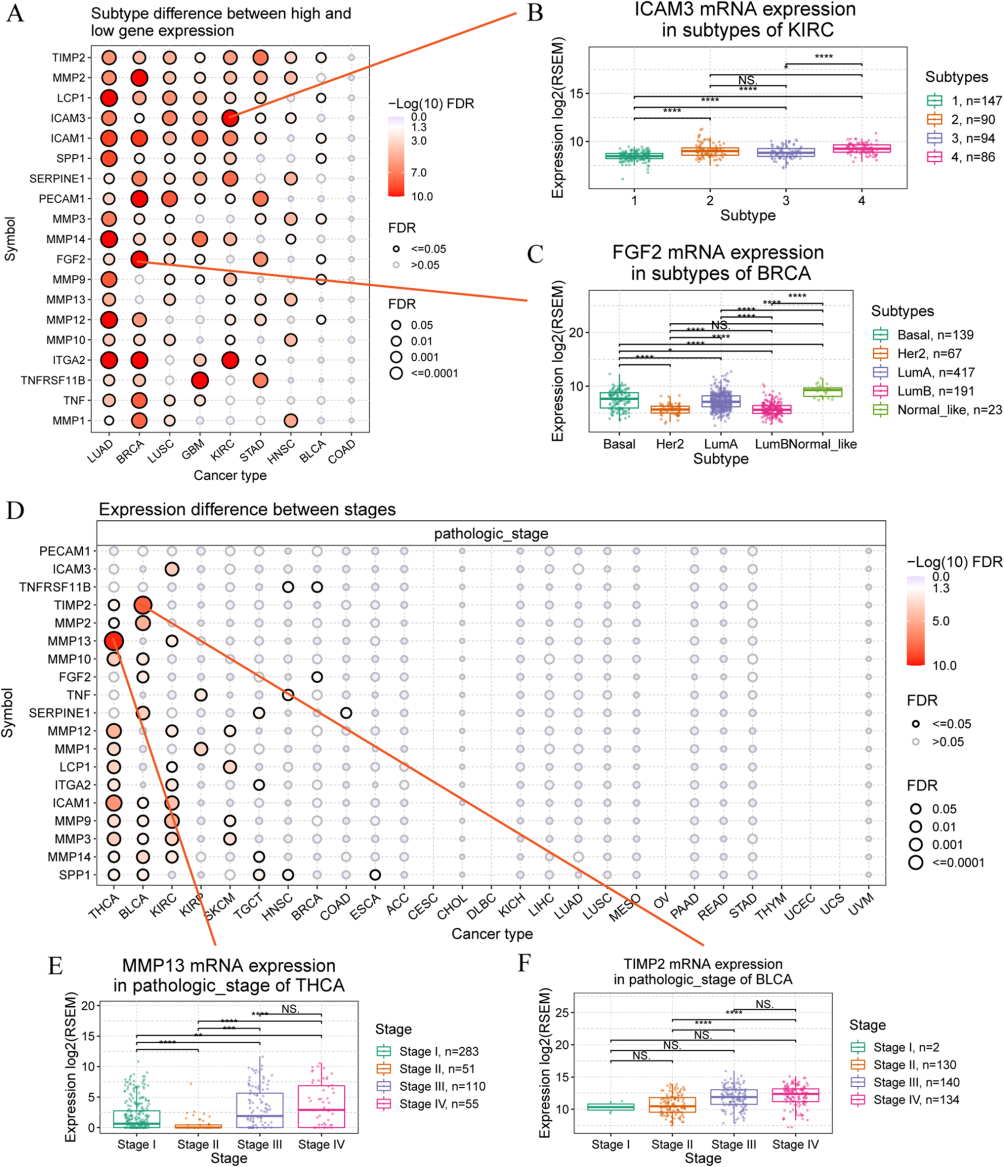
Genes set expression and subtype and stage analysis of extracellular-matrix-senescence-related genes (ECM-SRGs). **A** Subtype difference between high and low gene expression of ECM-SRGs in cancers. **B** ICAM3 mRNA expression in subtype of KIRC. **C** FGF2 mRNA expression in subtype of BRCA. **D** Expression difference between stages. **E** MMP13 mRNA expression in pathologic stage of THCA. **F** TIMP2 mRNA expression in pathologic stage of BLCA

We conducted a screening for clinically relevant genes influencing cancer subtypes. The results revealed significant differences in the expression of ECM-SRGs in patients with LUAD, BRCA, LUSC, GBM, KIRC, STAD, HNSC, and BLCA compared to other genes (FDR ≤ 0.05, Fig. 4A and Table S10). Additionally, we noted variations in ICAM3 expression among patients with KIRC subtypes (Fig. 4B) and FGF2 expression among patients with BRCA subtypes (Fig. 4C).

We conducted a screening for clinically relevant genes influencing cancer stages. The results indicated significant differences in the expression of ECM-SRGs among patients in different stages (from stage I to IV) of THCA, BLCA, KIRC, KIRP, SKCM, and TGCT (*FDR* ≤ *0.05*, Fig. 4D and Table S11). Such as *MMP13* expression in pathologic stages of THCA (Fig. 4E) and *TIMP2* expression in pathologic stages of BLCA (Fig. 4F).

**Fig. 5:**
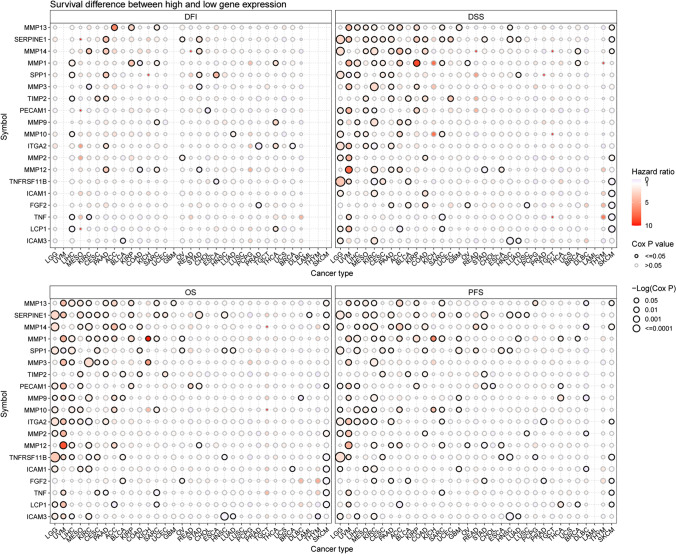
Survival difference between high and low gene expression in 33 cancers of extracellular-matrix-senescence-related genes. Red points represents worse survival of the high expression group, light blue points represent worse survival of the low expression group. The size of the point represents the statistical significance, where the larger the dot size, the higher the statistical significance

Furthermore, survival outcomes varied between groups with high and low ECM-SRG expression. Significant correlations were observed between ECM-SRGs and the survival (DFI, DSS, OS, and PFS) of patients (*Cox P* < *0.05*, Fig. 5 and Table S12). Then, a significant correlation between ECM-SRGs and the survival (OS and DFS) of patients was also identified in GEPIA2 (*P* < *0.05*, Fig. S2). These findings suggest that cancer may be affected by abnormal ECM-SRG expression.

### CNVs in ECM-SRGs

**Fig. 6:**
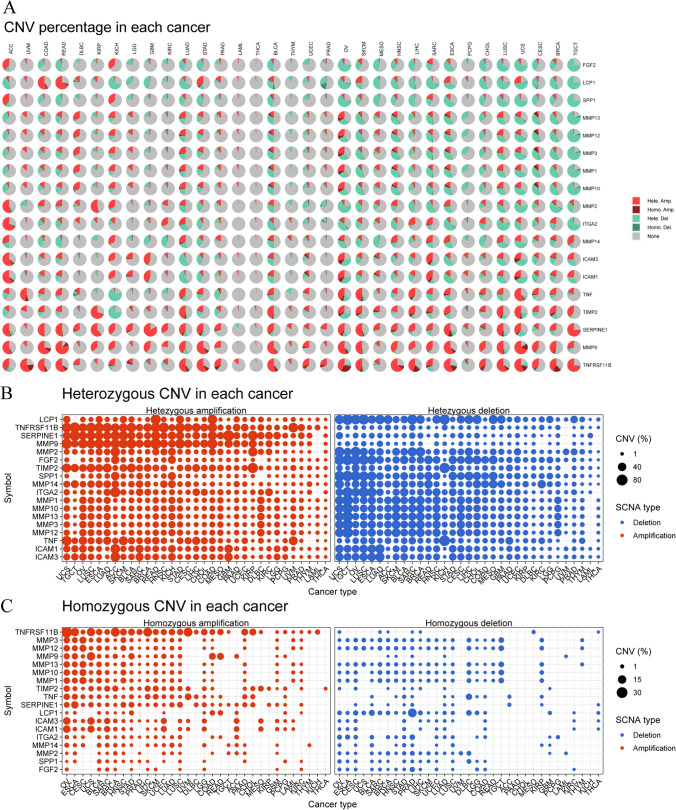
copy number variation (CNV) distribution in 33 cancers of extracellular-matrix-senescence-related genes. **A** CNV distribution. CNV pie chart showing the combined heterozygous/homozygous CNV of each gene in each cancer. A pie chart representing the proportion of different types of CNV of one gene in one cancer, and different colors represent different types of CNV. *Hete Amp* heterozygous amplification; *Hete Del* heterozygous deletion; *Homo Amp* homozygous amplification; *Homo Del* homozygous deletion; *None* no CNV. CNV profile showing the percentage of heterozygous CNVs (**B**) and Homozygous CNVs (**C**), including the percentage of amplification and deletion for inflammatory aging clock-related genes in 33 cancers. Only genes with > 1% CNV in a given cancer are shown as a point on the Figure

**Fig. 7:**
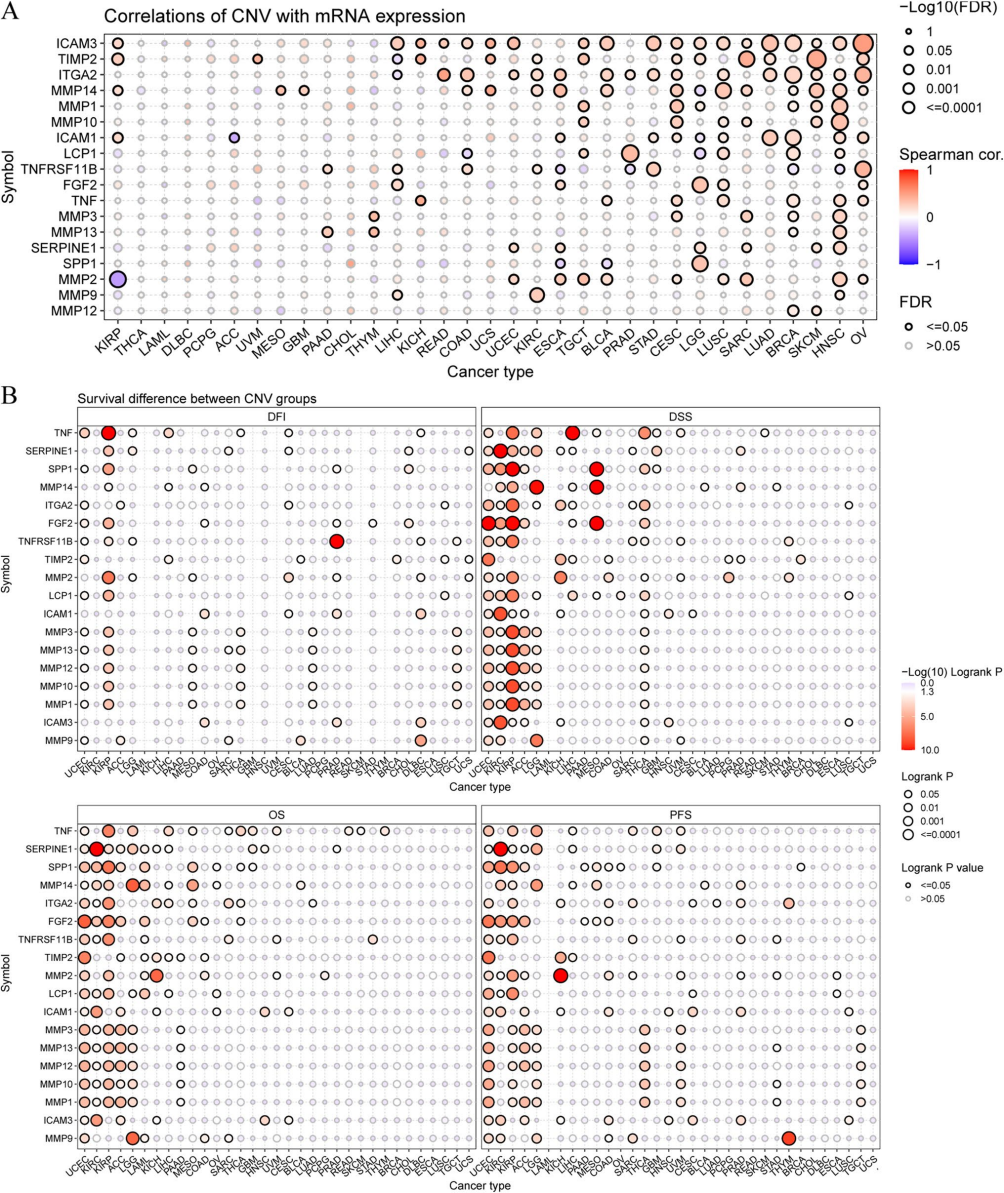
Copy number variation (CNV) correlation with mRNA expression and survival difference between CNV groups in 33 cancers of extracellular-matrix-senescence-related genes. **A** CNV correlation with mRNA expression. The association between paired mRNA expression and CNV percentage in samples was based on a Spearman’s product moment correlation coefficient. The size of the point represents the statistical significance, where the bigger the dot size, the higher the statistical significance. FDR, false discovery rate. **B** Survival difference between CNV groups. Red points represents worse survival of the hyper-CNV group, light blue points represent worse survival of the hypo-CNV group. The size of the point represents the statistical significance, where the larger the dot size, the higher the statistical significance

To identify CNVs in ECM-SRGs, we analyzed patient CNV data from TCGA. The CNV distribution pie chart indicated that the most commonly observed CNVs in patients were heterozygous amplifications and deletions (Fig. 6A and Table S13). Furthermore, genomic alterations were predominantly amplifications and deep deletions in cBioPortal (Fig. S3). The bubble plot will be filled with bubbles because heterozygous amplifications and deletions of ECM-SRGs were frequently observed in various cancers, as indicated by CNV percentage analyses (*P* < *0.05*, Fig. 6B). In most cancers, homozygous evaluation of ECM-SRGs showed both amplifications and deletions (*P* < *0.05*, Fig. 6C). Additionally, there was a positive association between ECM-SRG expression and CNV in patients with OV, HNSC, SKCM, BRCA, LUAD, SARC, LUSC, LGG, CESC, and others. However, the results revealed a negative correlation between MMP2 expression and CNV in KIRP patients and between ICAM1 expression and CNV in ACC patients (*P* < *0.0001*, Fig. 7A and Table S14). These results suggested that abnormal ECM-SRGs expression resulting from CNVs may be involved in the initiation and progression of cancer. Survival analysis (DFI, DSS, OS, and PFS) revealed a connection between high CNVs in ECM-SRGs and poor patient survival in various malignancies (*P* < *0.05*, Fig. 7B and Table S15).

### Analysis of methylation levels of ECM-SRGs

**Fig. 8:**
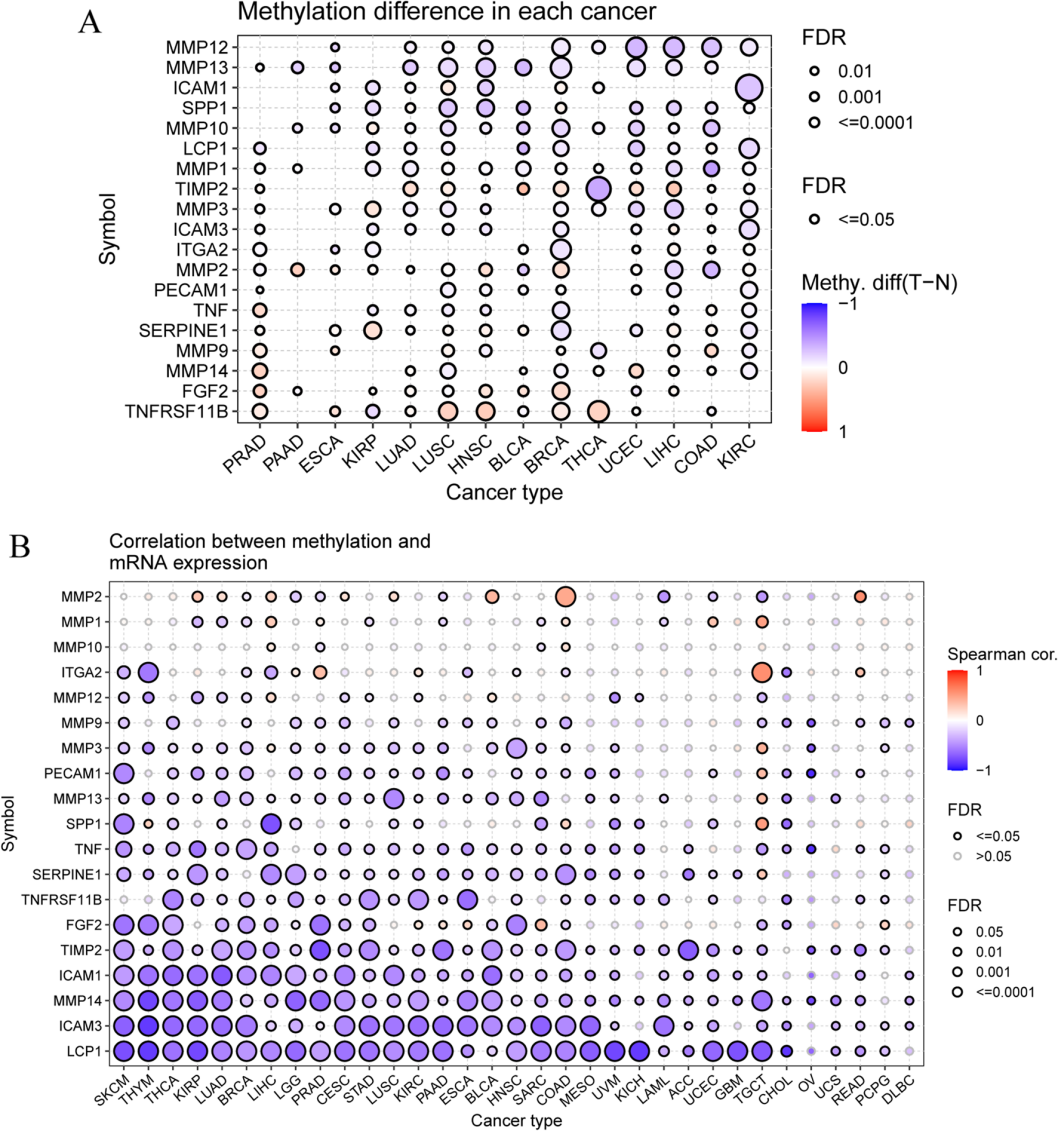
Methylation of extracellular-matrix-senescence-related genes (ECM-SRGs). **A** Differential methylation in ECM-SRGs between tumor and normal samples in each cancer. Blue points represent decreased methylation in tumors and red points represent increased methylation in tumors, where the darker the color, the larger the difference of methylation level. **B** Correlation between methylation and mRNA expression. Blue points represent a negative correlation and red points represent a positive correlation, where the darker of color, the higher the correlation. All the FDR of gene and cancer types were less than 0.05 in the Fig. FDR, false discovery rate

**Fig. 9:**
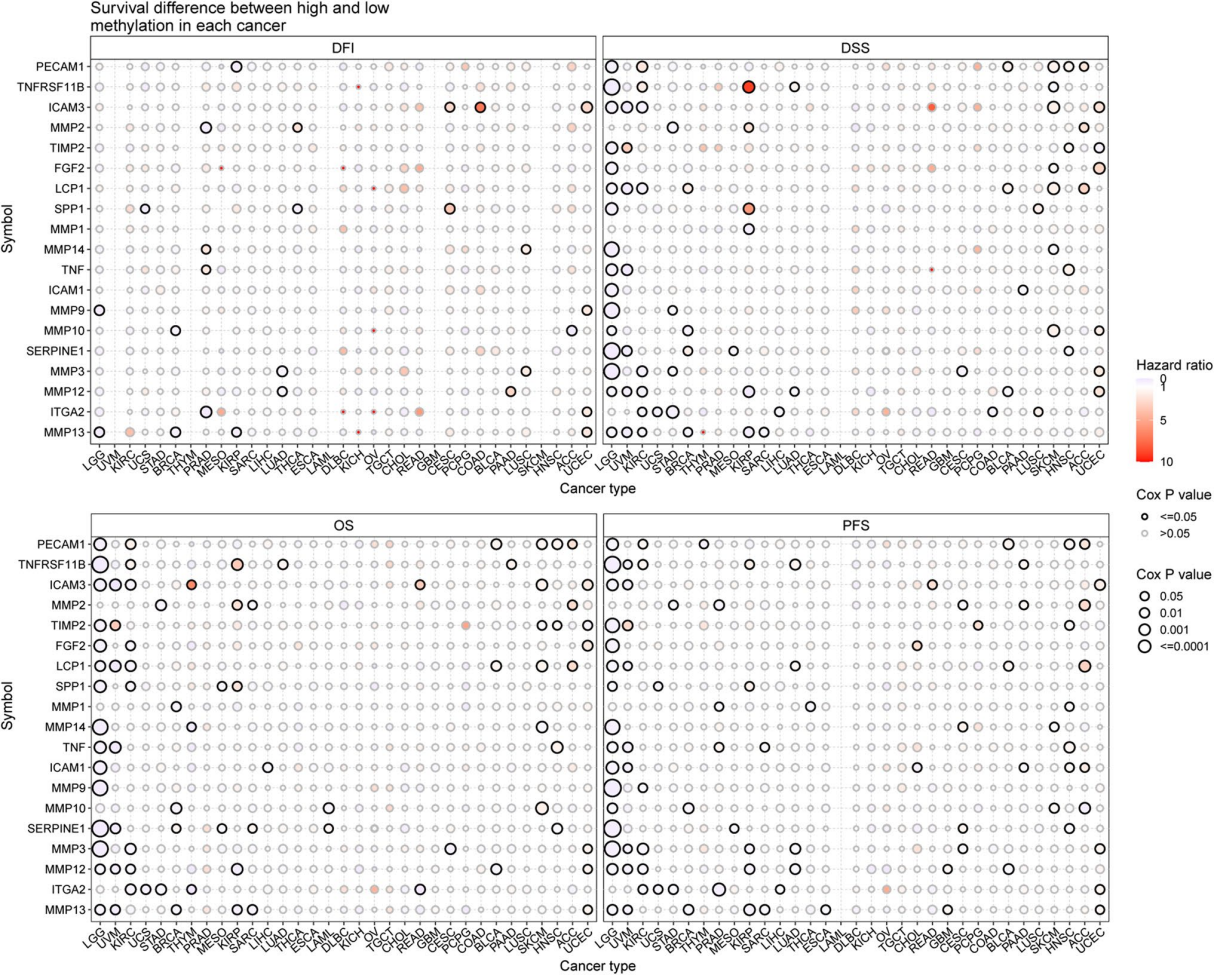
Survival difference between samples with extracellular-matrix-senescence-related genes with high and low methylation. Red points represents worse survival of the hypermethylation group, light blue points represent worse survival of the hypomethylation group. The size of the point represents the statistical significance, where the larger the dot size, the higher the statistical significance

We investigated epigenetic regulation by examining the methylation status of ECM-SRGs. The methylation status of ECM-SRGs exhibited significant heterogeneity among patients (Fig. 8A). The findings indicated a prevalence of hypomethylation in ECM-SRGs among patients with various cancers (KIRC, COAD, LIHC, UCEC, THCA, BRCA, etc.), and hypermethylation of TNFRSF11B among patients with PRAD, ESCA, LUSC, HNSC, BRCA, and THCA (*FDR* ≤ *0.05*, Fig. 8A and Table S16). The relationship between gene expression and methylation status was subsequently investigated. The results revealed a negative correlation between methylation and the expression of most ECM-SRGs, especially LCP1 expression, in patients with various malignancies (SKCM, THYM, THCA, KIRP, LUAD, BRCA, LIHC, LGG, etc.). However, a positive correlation was observed between MMP2 methylation and expression in patients with COAD, READ, BLCA, and ITGA2 methylation and expression in patients with TGCT (*FDR* ≤ *0.05*, Fig. 8B and Table S17). Survival analysis (DFI, DSS, OS, and PFS) revealed a correlation between ECM-SRGs hypomethylation and poor patient survival in various malignancies (*Cox P* < *0.05*, Fig. 9 and Table S18).

### ECM-SRG somatic mutations

**Fig. 10:**
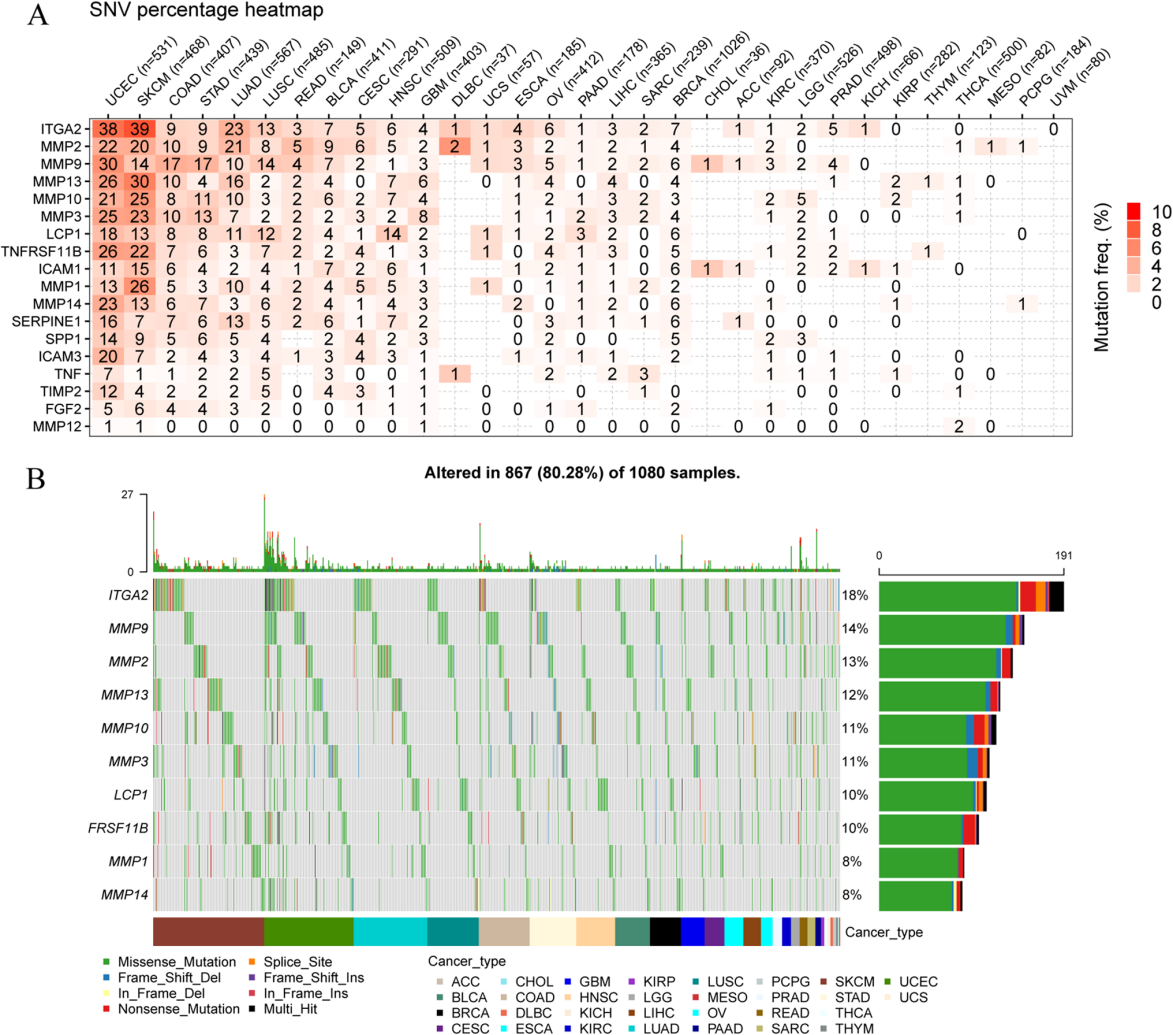
Single nucleotide variation (SNV) frequency and variant types of extracellular-matrix-senescence-related genes (ECM-SRGs). **A** Mutation frequency of ECM-SRGs. Numbers represent the number of samples that have the corresponding mutated gene for a given cancer. ‘0’ indicates that there was no mutation in the gene coding region, and no number indicates there was no mutation in any region of the gene. **B** SNV oncoplot. An oncoplot showing the mutation distribution of ECM-SRGs and a classification of SNV types

To assess the frequency of gene variants in each cancer subtype, we investigated SNPs in ECM-SRGs. LUAD, UCEC, COAD, STAD, SKCM, and LUSC patients exhibited an SNV frequency in ECM-SRGs ranging from 1 to 39%, as illustrated in Fig. 10A and Table S19. The frequency of SNVs in the regulatory genes was 80.28% (867 out of 1080 patients, Fig. 10B). Furthermore, missense mutations were the predominant type of SNPs in patients. The proportion of SNVs in the top 10 genes with mutations, ITGA2, MMP9, MMP2, MMP13, MMP10, MMP3, LCP1, FRSF11B, MMP1, and MMP14, was 18, 14, 13, 12, 11, 11, 10, 10, 8, and 8%, respectively. Patients with LUAD, UCEC, SKCM, and LUSC exhibited a higher frequency of SNVs in the regulatory genes (Fig. 10B). For patients with certain malignancies, survival analysis (DFI, DSS, OS, and PFS) demonstrated a significant difference in SNVs between mutant and WT ECM-SRGs (*Cox P* < *0.05*, Fig. S4 and Table S20).

### Pathway activity analysis

**Fig. 11:**
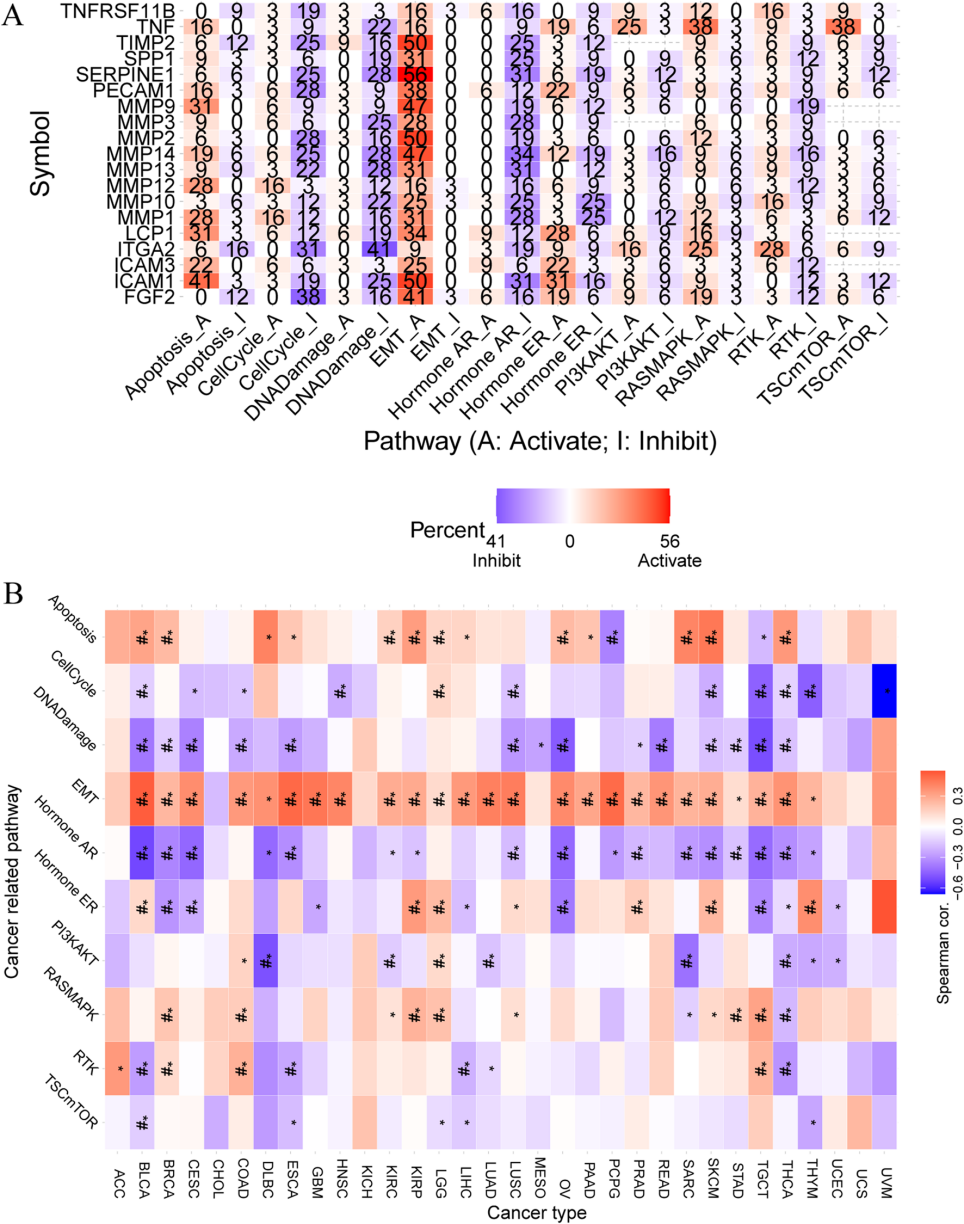
The cancer related pathway activity between extracellular-matrix-senescence-related genes (ECM-SRGs). **A** The combined percentage of the effect of ECM-SRGs on pathway activity. **B** Pathway activity of ECM-SRGs based on the GSVA score

The pathway activity analysis revealed a notable contribution of ECM-SRGs to cancer-related pathways, encompassing the cell cycle, apoptosis, PI3K/AKT, RAS/MAPK, RTK, and TSC/mTOR signaling pathways, EMT, hormone AR, and ER, as well as the response to DNA damage (Fig. 11A). These ECM-SRGs primarily played roles in activating EMT, apoptosis, and the RAS/MAPK signaling pathways, while inhibiting the cell cycle, hormone AR, and the response to DNA damage signaling pathways (*P* < *0.05*, Fig. 11A and Table S21). Subsequently, we assessed the pathway activity of ECM-SRGs based on the GSVA score, and the findings indicated the participation of ECM-SRGs in the activation of the EMT, apoptosis, and RAS/MAPK signaling pathways, as well as the suppression of the cell cycle, hormone AR, PI3K/AKT, and response to DNA damage signaling pathways (*P* < *0.05*, Fig. 11B). Furthermore, we conducted an analysis of the correlation between ECM-SRGs scores and 14 functional states in various tumors using CancerSEA. The results demonstrated the engagement of ECM-SRGs in activating EMT, apoptosis, angiogenesis, hypoxia, inflammation, and metastasis signaling pathways, while suppressing cell cycle, DNA repair, and DNA damage signaling pathways (*P* < *0.05*, Fig. S5). Thus, ECM-SRGs could modulate pathways related to cancers.

### Analysis of drug sensitivity

**Fig. 12:**
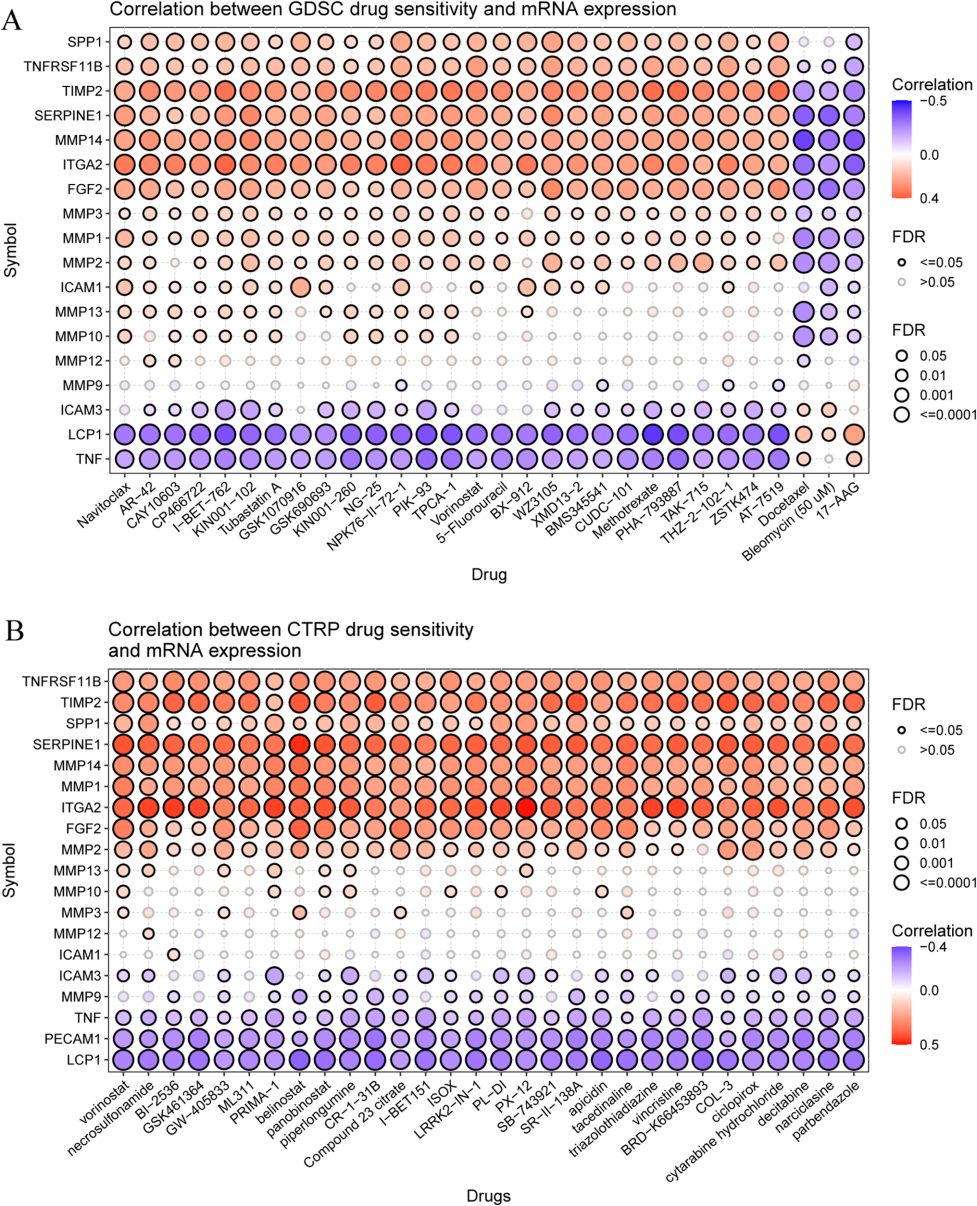
Correlation between (**A**) GDSC and (**B**) CTRP drug sensitivity and mRNA expression in pan-cancer. Spearman’s correlation represents how the gene expression correlates with a drug. A positive correlation means that a gene with high expression was resistant to a drug, and a negative correlation means that a gene with high expression was sensitive to a drug. *FDR* false discovery rate, *GDSC* Genomics of Drug Sensitivity in Cancer, *CTRP* Cancer Therapeutics Response Portal

Patient sensitivity to chemotherapy and targeted therapy can be influenced by genomic abnormalities. Thus, we explored the role of ECM-SRGs in mediating patient responses to chemotherapy and targeted therapy. Initially, we integrated data on gene expression in cancer cells and drug sensitivity from the GDSC. Subsequently, through Spearman's correlation analysis, we identified that the expression of TNF, LCP1, and ICAM3 was negatively correlated with drug sensitivity to compounds such as Navitoclax, AR-42, CAY10603, CP466722, I-BET-762, KIN001-102, Tubastatin A, GSK1070916, GSK690693, KIN001-260, NG-25, NPK76-II-72-1, PIK-93, TPCA-1, Vorinostat, 5-Fluorouracil, BX-912, WZ3105, XMD13-2, BMS345541, CUDC-101, Methotrexate, PHA-793887, TAK-715, THZ-2-102-1, ZSTK474, and AT-7519 (with a negative correlation with IC_50_ values). Conversely, resistance to Navitoclax, AR-42, CAY10603, CP466722, I-BET-762, KIN001-102, Tubastatin A, GSK1070916, GSK690693, KIN001-260, NG-25, NPK76-II-72-1, PIK-93, TPCA-1, Vorinostat, 5-Fluorouracil, BX-912, WZ3105, XMD13-2, BMS345541, CUDC-101, Methotrexate, PHA-793887, TAK-715, THZ-2-102-1, ZSTK474, and AT-7519 was associated with MMP14, ITGA2, FGF2, MMP3, MMP1, and MMP2 expression (positively correlated with IC_50_ values) (*FDR* ≤ *0.05*, Fig. 12A and Table S22).

Additionally, we integrated gene expression data from CTRP for cancer cell lines and their drug sensitivity. Through Spearman's correlation analysis, it was observed that drug sensitivity to Vorinostat, necrosulfonamide, BI-2536, GSK461364, GW-405833, ML311, PRIMA-1, belinostat, panobinostat, piperlongumine, CR-1-31B, Compound 23 citrate, I-BET151, ISOX, apicidin, 138A-II-SR, 743921-SB, 12-PX, DI-PL, 1-IN-LRRK2, tacedinaline, triazolothiadiazine, vincristine, BRD-K66453893, COL-3, ciclopirox, cytarabine hydrochloride, decitabine, narciclasine, and parbendazole was negatively correlated with LCP1, PECAM1, TNF, MMP9, and ICAM3 expression (with a negative correlation with IC_50_ values). In contrast, resistance to drugs such as Vorinostat, necrosulfonamide, BI-2536, GSK461364, GW-405833, ML311, PRIMA-1, belinostat, panobinostat, piperlongumine, CR-1-31B, Compound 23 citrate, I-BET151, ISOX, LRRK2-IN-1, apicidin, 138A-II-SR, 743921-SB, 12-PX, DI-PL, tacedinaline, triazolothiadiazine, vincristine, BRD-K66453893, COL-3, narciclasine, decitabine, cytarabine hydrochloride, ciclopirox, and parbendazole was positively correlated with TNFRSF11B, TIMP2, SPP1, SERPINE1, MMP14, MMP1, ITGA2, FGF2, and MMP2 expression (with a positive correlation with IC_50_ values) (*FDR* ≤ *0.05*, Fig. 12B and Table S23).

These findings suggest that aberrant expression of ECM-SRGs may serve as a mediator of resistance to both chemotherapy and targeted therapy.

## Discussion

During tumorigenesis, multiple alterations accumulate in cells over time. Recent studies have highlighted a significant increase in the levels of certain inflammatory components with age, particularly in cancer contexts [32–34]. This suggests a significant link between aging, inflammation, and cancer initiation. While the impact of aging-related dysregulation on cellular machinery in carcinogenesis is well-known, we often overlook the role of the extracellular matrix (ECM) and other microenvironmental changes. Notably, aged rats exhibit a higher tumor incidence after neoplastic transformation of their liver epithelial cells compared to young rats [18]. This points to the critical role of the aged microenvironment in tumor onset and progression. However, it remains unclear how aging in the ECM influences cancer development and spread. Therefore, investigating ECM-SRGs (Extracellular Matrix-Related Signaling Genes) could enhance our understanding of cancer and reveal potential therapeutic targets. To address this, we collected multi-omics profiling data and conducted a comprehensive systematic study of ECM-SRGs in over 10,000 patients with 33 cancer types, assessing more than 750 small molecule drugs and 24 immune cell types. This analysis identified a total of 19 ECM-SRGs. Notably, one study has already demonstrated a correlation between aged breast ECM and breast carcinoma risk [35]. Additionally, MMP2 expression appears influenced by the aged and stiffer matrix, potentially promoting migration and invasion [36]. However, we lack comprehensive studies on the applications of ECM-SRGs in cancer. Furthermore, the genetic, immune, and clinical characteristics of ECM-SRGs in the context of these 33 cancers have not been assessed.

Our results reveal a significant correlation between ICI and the GSVA-ES of ECM-SRGs across these 33 cancer types. Furthermore, we observe a positive correlation between the GSVA-ES of ECM-SRGs and patient Infiltration Scores across various cancers. Importantly, patients with SNV and CNV of ECM-SRGs in diverse malignancies exhibit substantial variations in ICI. These findings indicate that aberrant ECM-SRG expression and alterations in the immune microenvironment play a role in tumorigenesis, cancer progression, diagnosis, prognosis, and therapeutic outcomes. Recent investigations also support the involvement of key ECM-SRG components in modulating ICI [17, 37–43].

Our analyses highlight a high CNV frequency in ECM-SRGs, with CNV and ECM-SRG expression showing a positive correlation. Moreover, a high CNV frequency in ECM-SRGs in various cancers is associated with poorer patient survival, suggesting that CNV may affect ECM-SRG expression and contribute to tumorigenesis and survival. Our examination of epigenetic changes reveals that aberrant hypomethylation can increase ECM-SRG expression, which correlates with worse patient survival across multiple cancers. Additionally, our analysis shows a high frequency of SNV in ECM-SRGs, with a positive correlation between SNV and ECM-SRG expression. This high frequency of SNV in ECM-SRGs also correlates with poorer patient survival in several cancer types, underscoring the potential role of SNV in ECM-SRG dysregulation and its impact on tumorigenesis.

Pathway analysis uncovers that these ECM-SRGs can regulate pathways associated with cancer, including the activation of Epithelial-Mesenchymal Transition (EMT), apoptosis, and the RAS/MAPK signaling pathway [44–46]. These findings indicate that ECM-SRGs collectively form a network of pathways related to cancer, which can influence cancer progression and improve patient survival.

Notably, these ECM-SRGs are found to activate EMT, apoptosis, and the RAS/MAPK signaling pathways while suppressing the cell cycle, hormone AR (Androgen Receptor), and the response to DNA damage signaling pathways (*P* < *0.05*, Fig. 11A). Furthermore, an analysis of pathway activity based on the GSVA score reveals the involvement of ECM-SRGs in activating EMT, apoptosis, and the RAS/MAPK signaling pathway while suppressing the cell cycle, hormone AR, PI3K/AKT, and the response to DNA damage signaling pathway (*P* < *0.05*, Fig. 11B). Therefore, ECM-SRGs can modulate pathways closely related to cancer. The identification of potential drugs that can modulate these 19 ECM-SRGs leads us to hypothesize that targeting ECM-SRGs could be an effective strategy for treating cancer patients. However, further research is essential to understand the mechanisms through which these drugs affect ECM-SRG expression and cancer progression.

Our results unveil potential mechanisms underlying the involvement of ECM-SRGs in cancer development and their influence on the immune microenvironment. These findings also highlight the correlation between common ECM-SRGs and pathways related to cancer. While our study presents valuable insights, it has some limitations. One significant drawback is the challenge of dynamically analyzing ECM-SRG expression in paired cancer tissues at different timelines. While we searched TCGA for patient transcriptomic data to investigate the relationship between ECM-SRGs and cancer, these results must be validated experimentally. Nonetheless, our research provides novel insights into the regulation of ECM-SRGs in cancer. Moreover, the observed variations in genetics, epigenetics, expression levels, and pathway correlations may lead to differences in pharmacological effects, patient responses to therapy, and overall patient survival. Hence, a comprehensive analysis of cancer heterogeneity and personalized therapy is imperative for further understanding and effective management of this complex disease.

## Conclusion

In conclusion, our comprehensive evaluation of the genomes and immunogenomics of ECM-SRGs, along with an analysis of their clinical features across 33 solid tumors, has provided valuable insights into the intricate relationship between ECM-SRGs and tumorigenesis. These findings underscore the potential significance of ECM-SRGs in understanding and treating various cancer types. By shedding light on the genetic, epigenetic, and immune factors associated with ECM-SRGs, our research offers a promising avenue for the clinical treatment of cancer. The knowledge gained from this study may lead to more targeted and effective approaches to cancer diagnosis, prognosis, and therapeutic interventions. Moving forward, further research in this direction should focus on translating these discoveries into practical clinical applications, ultimately improving the outcomes and quality of life for cancer patients.

### Supplementary Information

Supplementary file1 (TIF 2420 KB)

Supplementary file2 (TIF 2175 KB)

Supplementary file3 (TIF 860 KB)

Supplementary file4 (TIF 1577 KB)

Supplementary file5 (TIF 1240 KB)

Supplementary file6 (DOCX 16 KB)

Supplementary file7 (DOCX 17 KB)

Supplementary file8 (DOCX 21 KB)

Supplementary file9 (XLSX 924 KB)

Supplementary file10 (XLSX 312 KB)

Supplementary file11 (XLSX 806 KB)

Supplementary file12 (XLSX 915 KB)

Supplementary file13 (XLSX 33 KB)

Supplementary file14 (XLSX 10 KB)

Supplementary file15 (XLSX 21 KB)

Supplementary file16 (XLSX 58 KB)

Supplementary file17 (XLSX 151 KB)

Supplementary file18 (XLSX 44 KB)

Supplementary file19 (XLSX 35 KB)

Supplementary file20 (XLSX 82 KB)

Supplementary file21 (XLSX 23 KB)

Supplementary file22 (XLSX 40 KB)

Supplementary file23 (XLSX 164 KB)

Supplementary file24 (XLSX 24 KB)

Supplementary file25 (XLSX 50 KB)

Supplementary file26 (XLSX 268 KB)

Supplementary file27 (XLSX 202 KB)

Supplementary file28 (XLSX 401 KB)

Supplementary file29 (DOCX 16 KB)

## Data Availability

All data are incorporated into the article and its online supplementary material. All the data displayed in the present manuscript are available from the corresponding author upon reasonable request.

## Abbreviations

ECM-SRGs: Extracellular-matrix-senescence-related genes
GSVA: Gene set variation analysis
SNV: Single nucleotide variation
CNV: Copy number variation
GBM: Glioblastoma multiforme
ICI: Immune cell infiltration
GSEA: Gene-set enrichment analysis
DSS: Disease-specific survival
DFI: Disease-free interval
HRG: High-expression group
LRG: Low-expression group
HR: Hazard ratio
FC: Fold change
MG: Mutant group
WT: Wild-type
PAS: Pathway activity score
LAML: Acute myeloid leukemia
ACC: Adrenocortical carcinoma
BLCA: Bladder urothelial carcinoma
BRCA: Breast invasive carcinoma
CESC: Cervical squamous cell carcinoma and endocervical adenocarcinoma
CHOL: Cholangiocarcinoma
COAD: Colon adenocarcinoma
ESCA: Esophageal carcinoma
GBM: Glioblastoma
HNSC: Head and neck squamous cell carcinoma
KICH: Kidney chromophobe
KIRC: Kidney renal clear cell carcinoma
KIRP: Kidney renal papillary cell carcinoma
LGG: Lower grade glioma
LIHC: Liver hepatocellular carcinoma
LUAD: Lung adenocarcinoma
LUSC: Lung squamous cell carcinoma
DLBC: Lymphoid neoplasm diffuse large B-cell lymphoma
MESO: Mesothelioma
OV: Ovarian serous cystadenocarcinoma
PAAD: Pancreatic adenocarcinoma
PCPG: Pheochromocytoma and paraganglioma
PRAD: Prostate adenocarcinoma
READ: Rectum adenocarcinoma
SARC: Sarcoma
SKCM: Skin cutaneous melanoma
STAD: Stomach adenocarcinoma
TGCT: Testicular germ cell tumors
THYM: Thymoma
THCA: Thyroid carcinoma
UCS: Uterine carcinosarcoma
UCEC: Uterine corpus endometrial carcinoma
UVM: Uveal melanoma
