# Supplementary figures and images for: Pan-cancer analyses of senescence-related genes in extracellular matrix characterization in cancer
Source: Discov Oncol. 2023 Nov 20;14:208. doi: 10.1007/s12672-023-00828-7 (PMC10660488; doi:10.1007/s12672-023-00828-7)

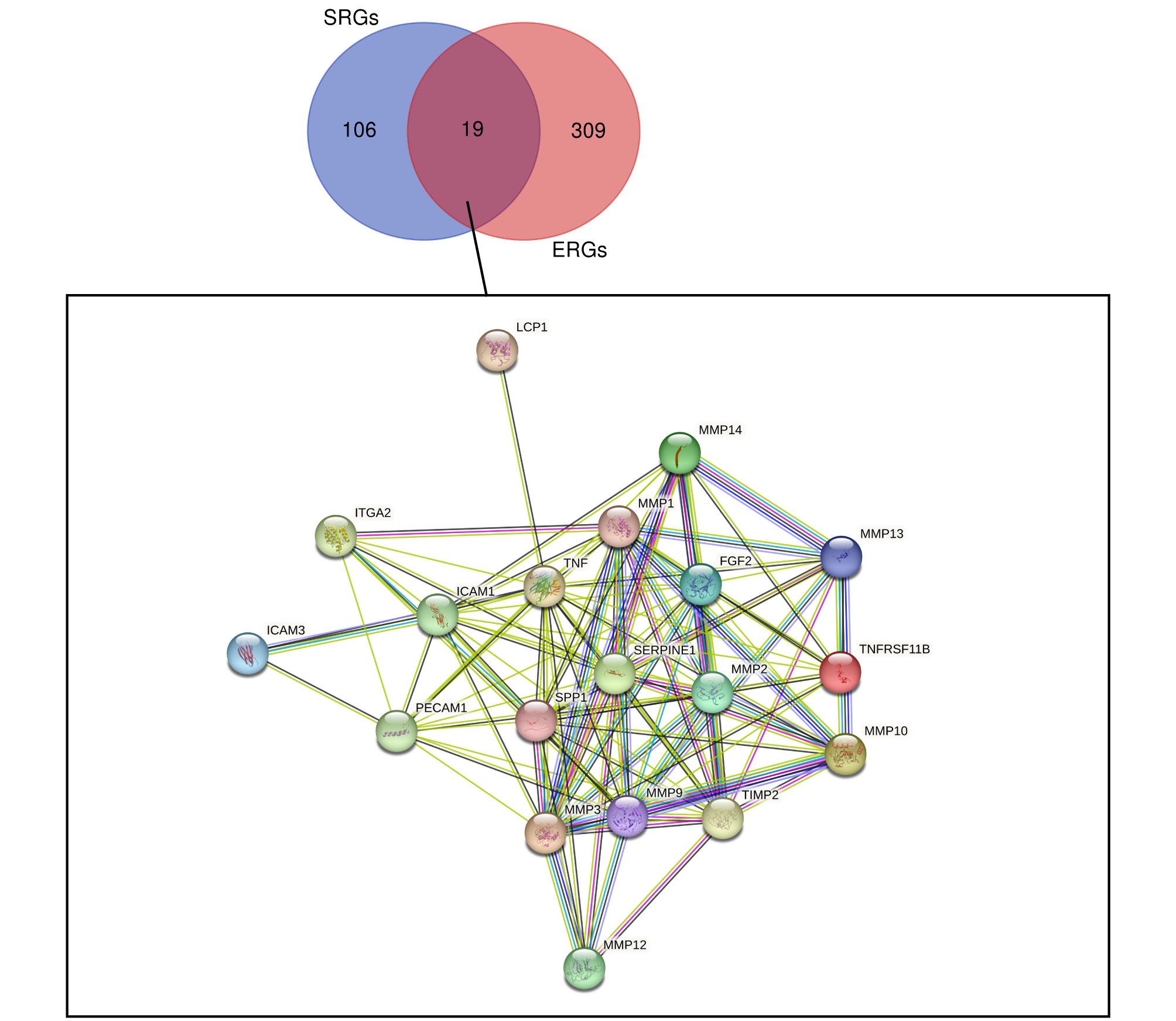

Supplement: Supplementary file 1 — Supplementary file1 (TIF 2420 KB) [file 12672_2023_828_MOESM1_ESM.tif]

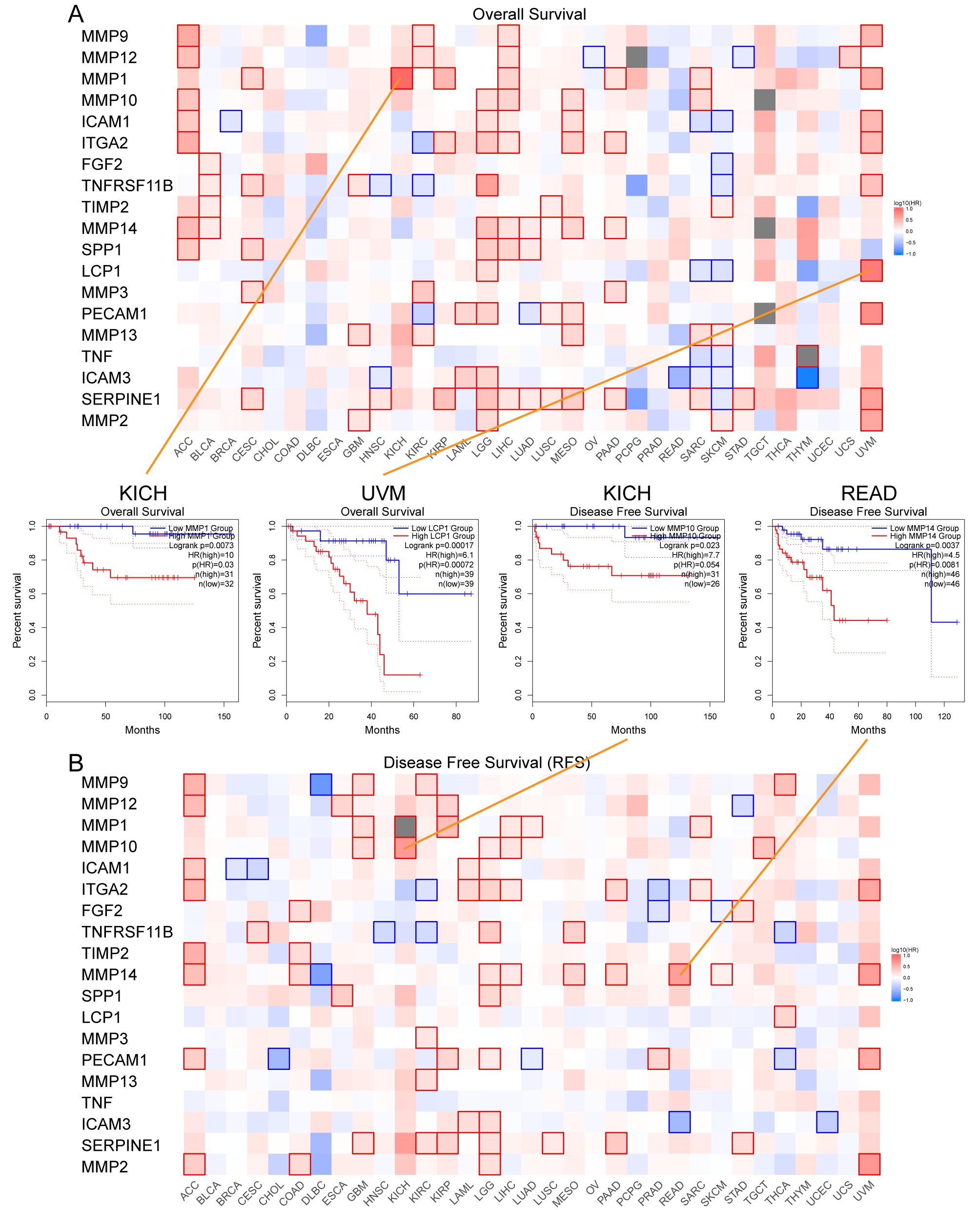

Supplement: Supplementary file 2 — Supplementary file2 (TIF 2175 KB) [file 12672_2023_828_MOESM2_ESM.tif]

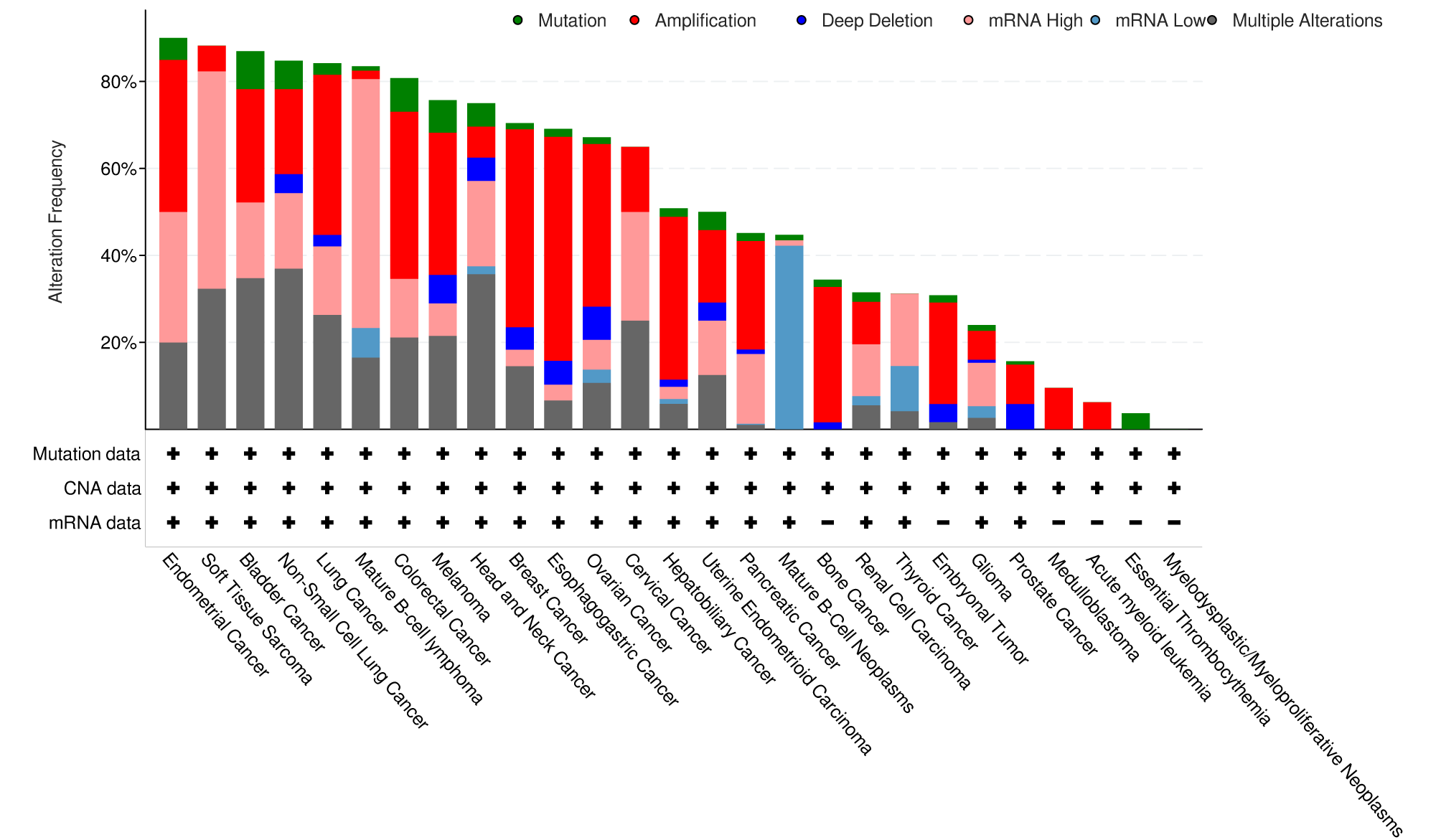

Supplement: Supplementary file 3 — Supplementary file3 (TIF 860 KB) [file 12672_2023_828_MOESM3_ESM.tif]

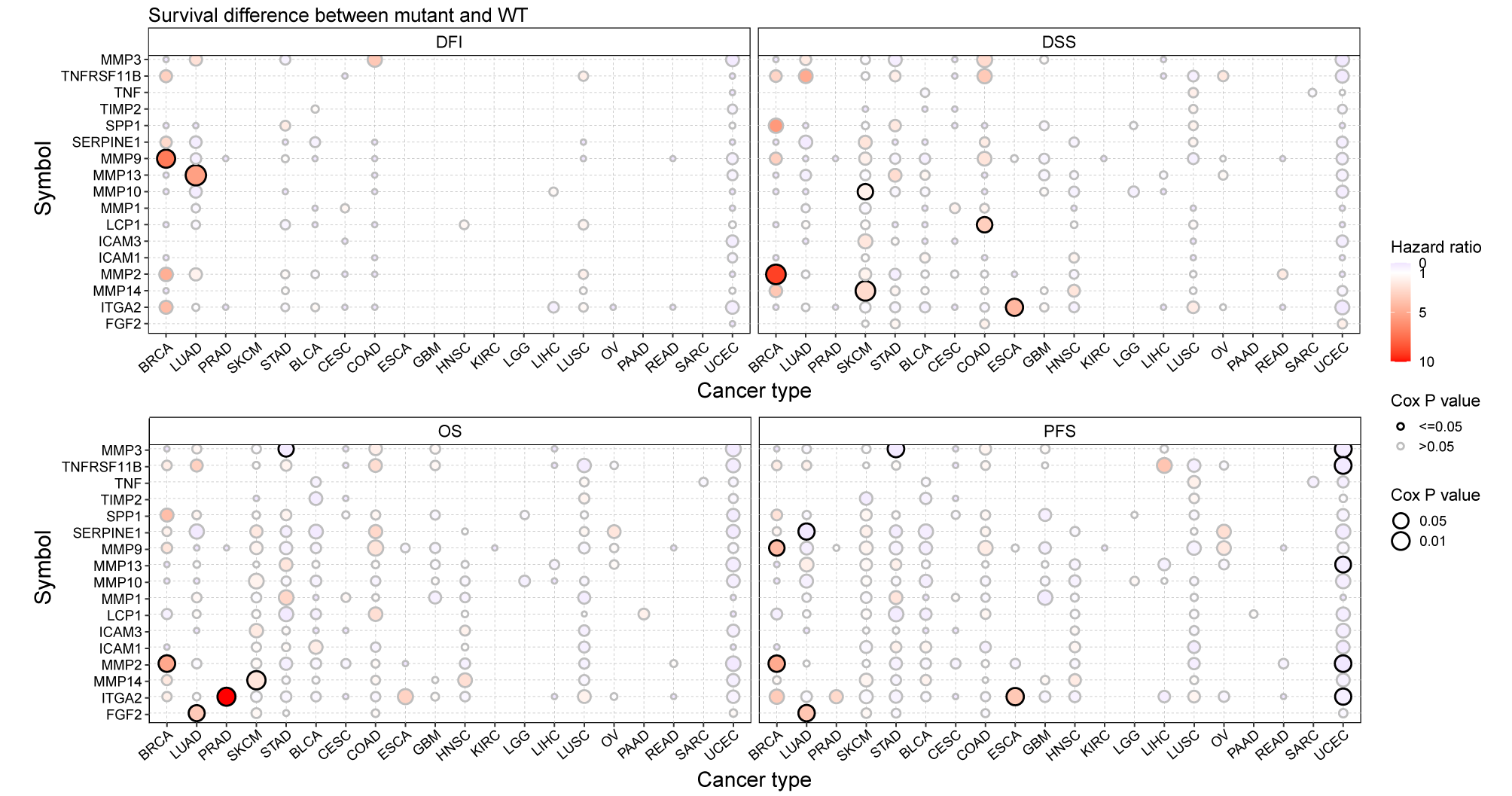

Supplement: Supplementary file 4 — Supplementary file4 (TIF 1577 KB) [file 12672_2023_828_MOESM4_ESM.tif]

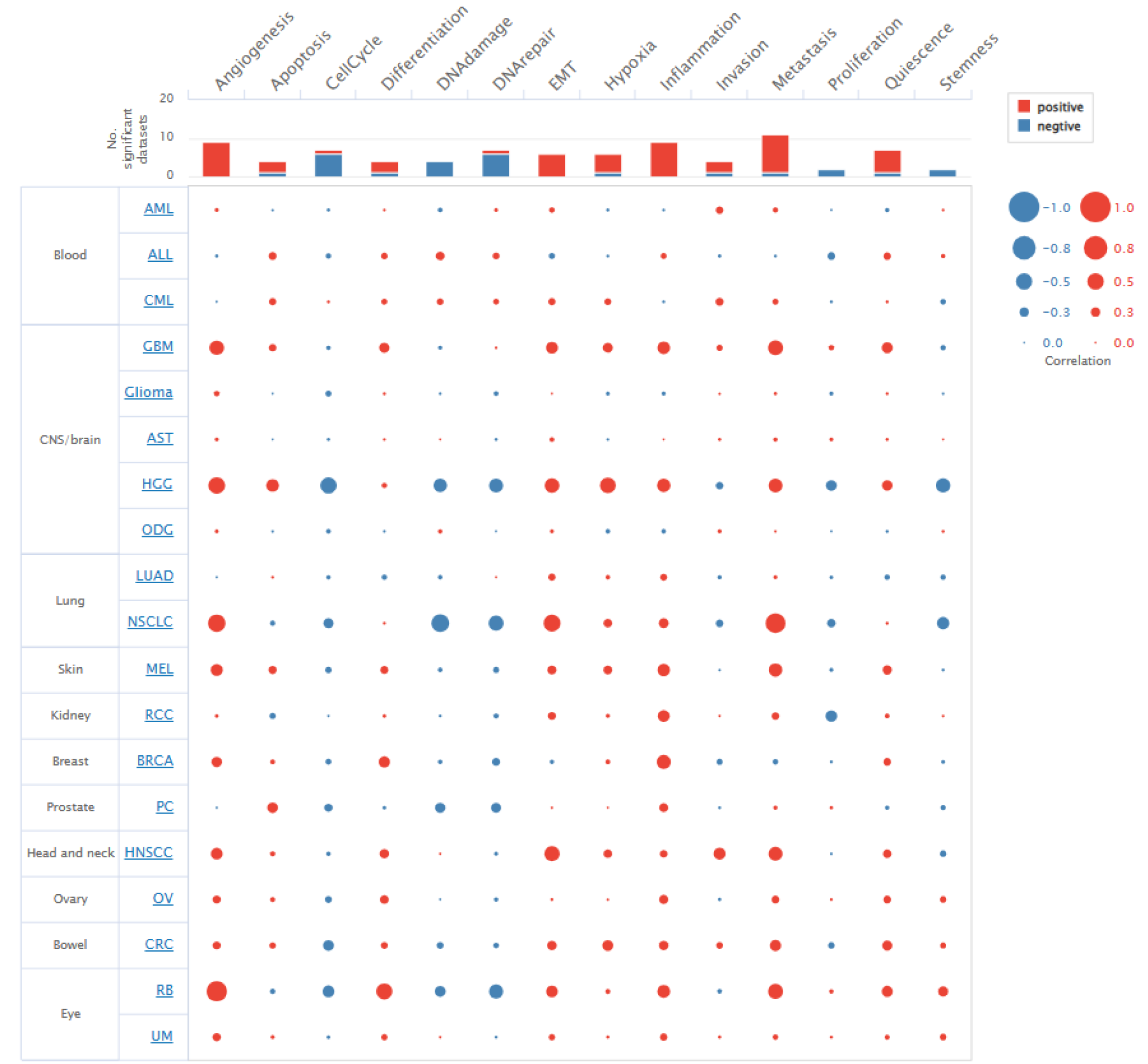

Supplement: Supplementary file 5 — Supplementary file5 (TIF 1240 KB) [file 12672_2023_828_MOESM5_ESM.tif]
