# Supplementary material for: Pan-cancer analyses of senescence-related genes in extracellular matrix characterization in cancer
Source: Discov Oncol. 2023 Nov 20;14:208. doi: 10.1007/s12672-023-00828-7 (PMC10660488; doi:10.1007/s12672-023-00828-7)
Supplement: Supplementary file 7 — Supplementary file7 (DOCX 17 KB) [file 12672_2023_828_MOESM7_ESM.docx]

**Table S2** samples of 33 cancer types

| **33 cancer types** | **Sample size** |
| --- | --- |
| Acute myeloid leukemia (LAML) | n = 191 |
| adrenocortical carcinoma (ACC) | n = 92 |
| bladder urothelial carcinoma (BLCA) | n = 464 |
| breast invasive carcinoma (BRCA) | n = 1218 |
| cervical squamous cell carcinoma and endocervical adenocarcinoma (CESC) | n = 331 |
| cholangiocarcinoma (CHOL) | n = 46 |
| colon adenocarcinoma (COAD) | n = 458 |
| esophageal carcinoma (ESCA) | n = 212 |
| glioblastoma (GBM) | n = 596 |
| head and neck squamous cell carcinoma (HNSC) | n = 598 |
| kidney chromophobe (KICH) | n = 112 |
| kidney renal clear cell carcinoma (KIRC) | n = 606 |
| kidney renal papillary cell carcinoma (KIRP) | n = 336 |
| lower grade glioma (LGG) | n = 551 |
| liver hepatocellular carcinoma (LIHC) | n = 450 |
| lung adenocarcinoma (LUAD) | n = 576 |
| lung squamous cell carcinoma (LUSC) | n = 554 |
| lymphoid neoplasm diffuse large B-cell lymphoma (DLBC) | n = 53 |
| mesothelioma (MESO) | n = 89 |
| ovarian serous cystadenocarcinoma (OV) | n = 582 |
| pancreatic adenocarcinoma (PAAD) | n = 207 |
| pheochromocytoma and paraganglioma (PCPG) | n = 189 |
| prostate adenocarcinoma (PRAD) | n = 573 |
| rectum adenocarcinoma (READ) | n = 170 |
| sarcoma (SARC) | n = 285 |
| skin cutaneous melanoma (SKCM) | n = 493 |
| stomach adenocarcinoma (STAD) | n = 450 |
| testicular germ cell tumors (TGCT) | n = 158 |
| thymoma (THYM) | n = 128 |
| thyroid carcinoma (THCA) | n = 588 |
| uterine carcinosarcoma (UCS) | n = 58 |
| uterine corpus endometrial carcinoma (UCEC) | n = 547 |
| uveal melanoma (UVM) | n = 81 |
