# Supplementary material for: Pan-cancer analyses of senescence-related genes in extracellular matrix characterization in cancer
Source: Discov Oncol. 2023 Nov 20;14:208. doi: 10.1007/s12672-023-00828-7 (PMC10660488; doi:10.1007/s12672-023-00828-7)
Supplement: Supplementary file 29 — Supplementary file29 (DOCX 16 KB) [file 12672_2023_828_MOESM29_ESM.docx]

**The tools used for the analysis based R:**

GSCA: <http://bioinfo.life.hust.edu.cn/GSCA/#/>

cBioPortal: <https://www.cbioportal.org/>

CancerSEA: <http://biocc.hrbmu.edu.cn/CancerSEA/>

[ImmuCellAI](http://bioinfo.life.hust.edu.cn/ImmuCellAI/#!/): <http://bioinfo.life.hust.edu.cn/ImmuCellAI/#!/>

**Raw data :**

TCGA: <https://portal.gdc.cancer.gov/>

UCSC Xena: <http://xena.ucsc.edu/>

Synapse: syn7824274; <https://www.synapse.org/#!Synapse:syn7824274>

TCPA: <https://tcpaportal.org/tcpa/index.html>

GDSC: <https://www.cancerrxgene.org/>

CTRP: <https://portals.broadinstitute.org/ctrp/>
